# Lateral-flow device for the diagnosis of invasive aspergillosis: a systematic review and diagnostic meta-analysis

**DOI:** 10.1186/s12879-025-10769-x

**Published:** 2025-03-20

**Authors:** Yuqing Fan, Xue Shang, Yan Wang, Yinghua zhang, Xiuxia Li, Kehu Yang, Haidi Lv, Kangle Guo

**Affiliations:** 1 Department of Infection Management/Department of urology, Gansu Provincial Hospital, No.204 Donggang West Road, Lanzhou, 730000 China; 2 School of Sociology, Huazhong University of Science and Technology, Wuhan, China; 3 Health Technology Assessment Center, School of public health, Lanzhou University, Lanzhou, China; 4 Evidence Based Medicine Center, School of Basic Medical Sciences, Lanzhou University, Lanzhou, China

**Keywords:** Lateral flow device, Invasive aspergillosis, Diagnostic meta-analysis

## Abstract

**Background:**

Early diagnosis of invasive aspergillosis (IA) can significantly enhance patient survival rates; however, accurately diagnosing IA remains a formidable challenge. Lateral flow device (LFD), as a non-invasive detection method, have been extensively investigated in numerous clinical studies. The objective of this study was to elucidate the diagnostic accuracy of LFD in detecting IA through a meta-analysis.

**Methods:**

The PubMed, Embase, and Web of Science database were searched to obtain clinical studies on the diagnosis of IA by LFD. A random-effects meta-analysis with a bivariate hierarchical model was used, the estimates and 95% confidence intervals (CI) were used to present pooled sensitivity, specificity, and summary receiver operating characteristic curves (SROC).

**Results:**

Twenty-five cohort or case-control studies were included. The pooled sensitivity of LFD in the diagnosis of IA was 0.67 (95% CI: 0.57–0.75), specificity was 0.90 (95% CI: 0.85–0.93), diagnostic odds ratio was 15.70 (95% CI: 9.69–25.44), the area under the SROC curve (AUC) was 0.87 (95% CI: 0.82–0.93). Subgroup analysis showed that the sensitivity of bronchoalveolar lavage fluid specimen was higher than serum specimen (0.72, 95% CI: 0.67–0.78 vs. 0.49, 95% CI: 0.41–0.56), bronchoalveolar lavage fluid specimens also have higher diagnostic accuracy (AUC = 0.89).

**Conclusions:**

LFD is an effective technique for the detection of IA infection, but attention should be paid to the influence of specimen source on the accuracy of this technique.

**Supplementary Information:**

The online version contains supplementary material available at 10.1186/s12879-025-10769-x.

## Introduction

Invasive aspergillosis (IA) is a life-threatening fungal disease caused by *Aspergillus spp.*, these *Aspergillus* species are widely found throughout the world. Among them [1], *Aspergillus fumigatus* is the primary etiological agent responsible for IA, a severe disseminated fungal disease characterized by high morbidity and mortality in severely immunocompromised individuals [2]. These include individuals with hematological malignancies, recipients of stem cell and organ transplantation, as well as individuals with lung disease or viral/bacterial pulmonary infections [3–5]. While traditional risk factors for IA centered on hematological malignancies, emerging evidence establishes specific determinants for COVID-19-associated pulmonary aspergillosis (CAPA), particularly prolonged respiratory support (> 10 days), cumulative corticosteroid dose (> 600 mg prednisone-equivalent), and intensive care unit environmental exposures during pandemic surges [6]. In a Swedish study investigating IA among acute leukemia patients, researchers reported a 19% prevalence rate [7]. A French multicenter surveillance epidemiological study demonstrated a 15% incidence of IA in organ transplantation recipients, accompanied by a 45% mortality rate [8]. A 2022 systematic review analyzing IA epidemiology across African countries revealed prevalence rates as high 27%, with case fatality rates exceeding 60% [9].

Therefore, in order to reduce IA-related mortality among the infected population, a comprehensive approach comprising multiple steps (e.g., risk identification, primary prevention, diagnosis and treatment, etc.) is imperative [10]. Among various strategies (e.g., identification of susceptible populations, environmental control, early diagnosis, effective treatment, immunity enhancement, etc.), enhancing the diagnostic accuracy of IA plays a pivotal role in facilitating timely implementation of effective treatment measures and thereby improving patient survival rates [11]. In contemporary clinical practice, culture and microscopy continue to serve as the gold standard for diagnosing IA, however, the absence of positive cultures in blood or tissue often delays definitive diagnosis [12, 13]. With advancing technology, Galactomannan, polymerase chain reaction (PCR), and (1–3)-β-D-glucan (BDG) assays show advantages in diagnosing IA, though they retain limitations. For instance, according to a 2016 U.S. guideline [14], Galactomannan detection in serum and bronchoalveolar lavage fluid (BALF) is recommended as a validated biomarker assay for IA diagnosis in adults and children, but not in patients undergoing active antifungal therapy or prophylaxis [15]. PCR’s role in IA diagnosis remains contentious. Although some evidence supports combining PCR with antigen detection assays, other experts argue against its routine implementation [16]. Ultimately, the utilization of PCR is complicated by the necessity to combine it with other diagnostic methods and consider the clinical context [17].

IA diagnosis remains challenging due to the limited sensitivity of mycological culture and the inherent difficulty in performing histopathology on critically ill patients [10]. While resource-limited settings struggle with diagnostic access, this reality underscores the critical demand for accessible diagnostic solutions [11]. The *Aspergillus*-specific lateral flow device (LFD) is an emerging point-of-care diagnostic method for invasive IA compared with the earlier specimen culture, Galactomannan, and PCR [18]. It utilizes the mouse monoclonal antibody JF5, which specifically binds to protein epitopes on extracellular glycoprotein antigens that are continuously secreted during active growth of *Aspergillus fumigatus*, achieving serum antigen detection within 15 min [19]. Initial validation demonstrated 73% sensitivity and 90% specificity using BALF [18, 20], with recent technical optimizations significantly enhancing specificity [21]. In recent years, numerous clinical studies have investigated the diagnostic accuracy of LFD in IA, however, these studies lack integration and evidence-based research is still insufficient. Therefore, this study aims to systematically review and conduct a diagnostic meta-analysis to explore the practical value of LFD accuracy in diagnosing IA.

## Methods

This study employed a methodical review and meta-analysis approach, following the established guidelines outlined in the Preferred Reporting Items for a Systematic Review and Meta-analysis of Diagnostic Test Accuracy Studies: The PRISMA-DTA Statement [22] (Appendix table S1).

### Search strategy

The PubMed, Embase, Web of Science, Cochrane Library, and Scopus were searched by two independent investigators from database establishment to October 2024. The search terms were as follows: (invasive aspergillosis OR IA OR aspergillosis) AND (lateral-flow device OR LFD OR non-invasive assay OR point-of-care diagnosis).

### Inclusion and exclusion criteria

The inclusion criteria were: (a) study design was observational, including cohort study and case-control study; (b) participants included IA patients, high-risk IA, or control patients, all participants had previously been diagnosed by gold standard (e.g. European Organization for Research and Treatment of Cancer/Mycoses Study Group (EORTC/MSG)), including proven/probable/no IA; (c) in each study, the LFD method was used to diagnose all samples, the sources of these specimens included bronchoalveolar lavage fluid, serum, or sputum; (d) each study reported the sample size of the case and control groups under the gold standard, and the outcome of the LFD diagnosis was presented, including direct indicators (true positives, true negatives, false positives and false negatives) and indirect indicators (sensitivity and specificity).

Studies were excluded based on the following criteria: (a) insufficient data, the required values could not be calculated based on the information available in the study, (b) meta-analysis and systematic reviews, (c) animal studies, and (d) studies not in English.

### Literature selection and data extraction

The retrieved literature was imported into literature management software (EndNote v.8x), after removing duplicate studies, two authors independently screened the literature. During the evaluation process, if there was a difference between the two authors, a third authors was consulted to resolve the disagreement. The first step of screening was based on the title and abstract, and after excluding irrelevant studies, full texts were obtained for potentially eligible studies. Then, inclusion or exclusion was decided based on more detailed information.

During the data extraction phase, a pre-designed data extraction table was provided, two reviewers independently extracted information from eligible articles. The information extracted from the included studies was the name of first author, study design, year of publication, country or region of the study, characteristics of the study population (age, concomitant disease), sample size, specimen source, and gold standard.

### Risk of bias and quality of evidence assessment

The quality of studies was assessed using the revised quality assessment of diagnostic accuracy studies (QUADAS-2) tool and the standards for reporting diagnostic accuracy (STARD) tool [23]. The tool includes “risk of bias” and “applicability concerns” assessment, some items could be judged as ‘yes’, ‘no’, or ‘unclear’, while other domains could be classified as low risk (concerns), high risk (concerns), or unclear risk of bias (concerns) [24].

Certainty of evidence was evaluated based on the Grades of Recommendation, Assessment, Development and Evaluation (GRADE) [25]. In the evaluation process, five factors were taken into account: risk of bias, indirectness, inconsistency, imprecision, and publication bias. Each factor can be assessed as not serious (no downgrade), serious (one grade reduction), or very serious (two grades reduction). The overall level of evidence was initially high and decreased with increasing downgrades, other resulting levels included moderate, low, and very low. Finally, a tabular representation is provided to summarize the evidence profile [26].

### Statistical analysis

Patients were classified into the following three groups according to the EORTC/MSG criteria [27]: proven IA, probable IA, and no IA. If studies provided explicit reporting of true positives, true negatives, false positives, and false negatives in relation to LFD diagnoses, these values were directly utilized. In cases where direct reporting was absent, the four metrics were derived based on sample size along with reported sensitivity and specificity, finally two-by-two tables for each study were constructed. A random-effects meta-analysis with a bivariate hierarchical model was used, and the generated indicators were presented using estimates and 95% confidence intervals (CI). The indicators included pooled sensitivity, specificity, positive likelihood ratios (PLR, describes how many times more likely positive index test results are with the target condition than without), negative likelihood ratios (NLR, describes how many times less likely negative index test results were with the target condition than without) [28], and diagnostic odds ratio (DOR, describes how many times higher the odds of obtaining a positive test result in someone with the target condition are than in someone without the target condition) [29]. In addition, a layered summary receiver operating characteristic (SROC, giving a visual indication of variability and heterogeneity) curve and diagnostic accuracy (area under the curve, AUC) was provided [30]. Furthermore, pooled PLR values of > 10 and pooled NLR values of < 0.1 means a convincing diagnostic evidence, whereas strong diagnostic evidence was based on pooled PLR values of > 5 and NLR values of < 0.2.

The statistically significant heterogeneity was assessed using I^2^ statistics and explored the potential heterogeneity between studies. To better investigate the impact of different factors on the diagnosis, subgroup analysis was performed for different variables, such as study design (cohort vs. case-control) and sample type (BALF vs. serum). In addition, the generated funnel plot was used to test for publication bias (P value < 0.05 means the presence of bias). Stata 15.1 and Meta-Disc software were used for all statistical calculations.

## Results

### Results of the systematic literature search

**Fig. 1:**
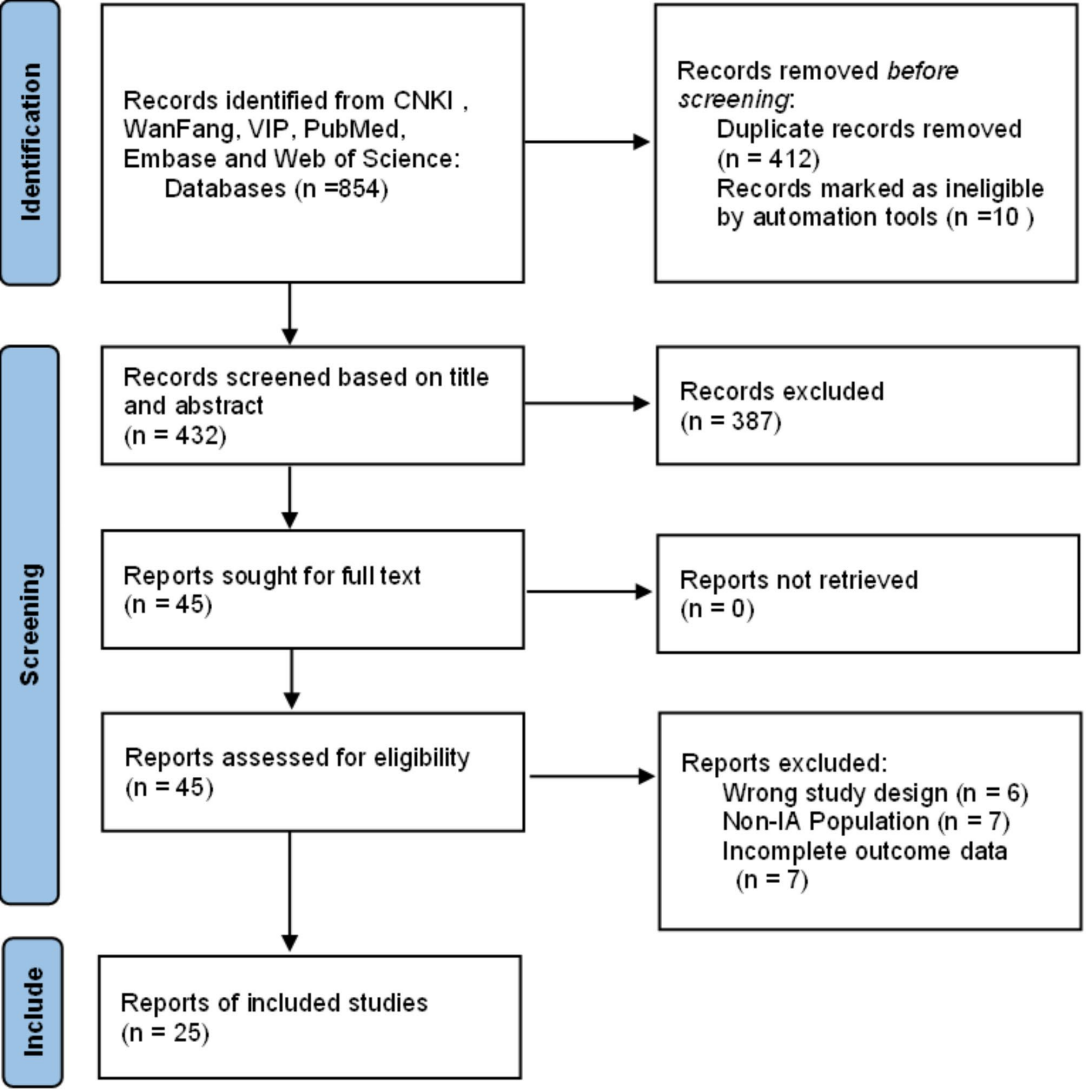
Flow chart of literature screening

854 studies were identified during the initial search, when duplicates were removed, 432 titles remained for screening (Fig. 1). Overall, 45 studies were selected for full text screening, finally 25 studies [31–55] were included in the systematic review and meta-analysis.

### Basic characteristics of included studies

**Fig. 2:**
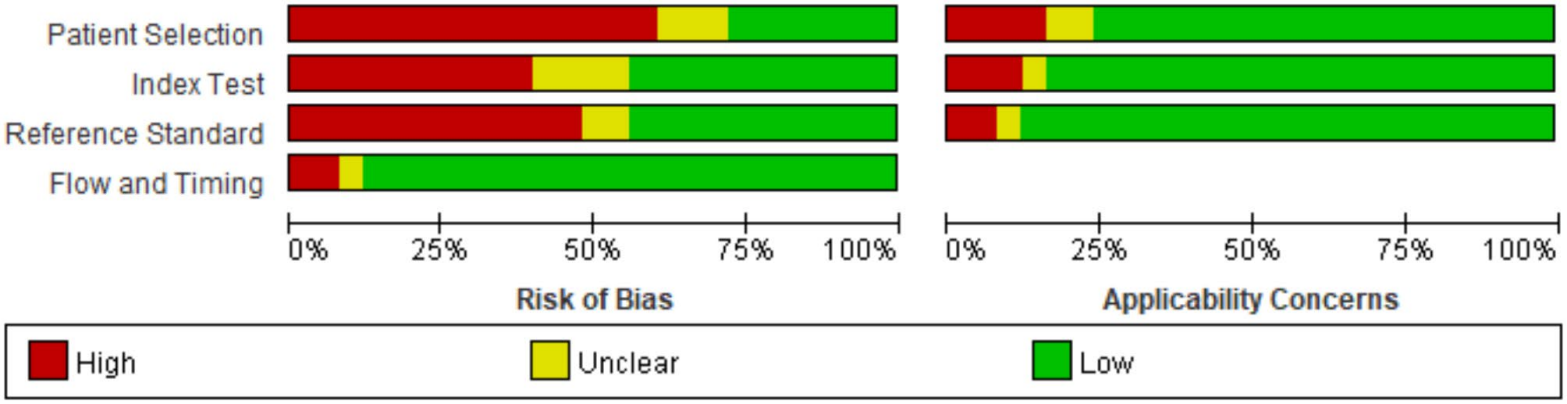
Overall quality assessment of the all included studies

All included studies were published between 2013 and 2024 and covered 10 countries. The study designs included cohort and case-control studies, and the conventional mean age of the population was >40 years. The majority of the enrolled population consisted of individuals with hematologic malignancies, while others included patients who had undergone hematopoietic stem-cell transplantation, were immunocompromised, or had respiratory conditions. Most studies reported BALF as the source of the samples, others included serum samples. The gold standard used in all studies was EORTC/MSG of different years (Table 1). The quality assessment results are represented in a bar chart (Fig. 2), including risk of bias (four domains) and applicability concerns (three domains), most studies (more than 75%) had a low risk of bias in terms of Flow and Timing and, while more studies had a high risk of bias in terms of Patient Selection (Appendix figure S1).

**Table 1:** Basic characteristics of the included studies

Study	Country	Design	Population	Sample size (tested)	Age	Definition	Specimen Source	Diagnostic standard
Wan 2024 [31]	China	Retrospective case-control	Cancer patients	191	Median (IQR): 60 (52–72)	Proven/probable vs. non-IA	Overall	EORTC/MSG-2020
Estella 2023 [32]	Spain	Prospective cohort	Severe SARS-CoV-2 pneumonia	160	Median (IQR): 65 (57–71)	Proven vs. non-IA	BALF	ECMM/ISHAM-2020
Xiao 2022 [33]	China	Prospective cohort	Respiratory diseases	127	Median (IQR): 66 (54–75)	Proven/probable vs. non-IA	Sputum	EORTC/MSG-2008
Hsiao 2022 [34]	China	Prospective cohort	Hematologic malignancies	104	Mean (Range): 54.5 (4–93)	Proven vs. non-IA	Serum	EORTC/MSG-2008
Chaturvedi 2022 [35]	India	Retrospective case-control	Hematological diseases	41	Median (range): 40 (21–65)	Proven vs. possible/probable IA	Serum	EORTC/MSG-2020
Aerts 2022 [36]	Belgium	Prospective cohort	Hematological diseases	41	Median: 56.2	Proven/probable vs. non-IA	Serum	EORTC/MSG-2020
Mercier 2021 [37]	Belgium	Prospective cohort	Hematological diseases	229	Median (IQR): 60 (51.5–66)	Proven/probable vs. non-IA	Serum	EORTC/MSG-2019
Scharmann 2020 [38]	Germany	Retrospective case-control	Cancer and lung diseases	200	Median: 61	Proven/probable vs. non-IA	BALF	EORTC/MSG-2019
Mercier 2020 [39]	Belgium	Retrospective case-control	Hematological diseases	192	Median (IQR): 64 (52–71)	Proven vs. non-IA	BALF	EORTC/MSG-2008
Takazono 2019 [40]	Japan	Retrospective case-control	Cancer and respiratory diseases	52	Mean ± SD: 66.5 ± 11.3	Proven vs. non-IA	Serum	NA
Mercier 2019 [41]	Belgium	Retrospective case-control	Hematological diseases	135	Median (IQR): 63 (52–71)	Proven vs. non-IA	BALF	EORTC/MSG-2008
Linder 2019 [42]	USA	Prospective cohort	Transplant patients	42	Mean ± SD: 52.7 ± 17.5	Proven/probable vs. non-IA	BALF	EORTC/MSG-2008
Jenks 2019 [43]	USA	Retrospective case-control	Hematological diseases	17	Median (IQR): 70 (24–78)	Proven/probable vs. non-IA	BALF	EORTC/MSG-2008
Heldt 2018 [44]	Austria	Prospective cohort	Hematological diseases	74	Median (Range): 55 (49–74)	Probable vs. non-IA	Serum	EORTC/MSG-2008
Castillo 2018 [45]	USA	Retrospective case-control	Transplant patients	114	Mean ± SD: 55.3 ± 16.8	Proven/probable vs. non-IA	BALF	EORTC/MSG-2008
Metan 2017 [46]	Turkey	Retrospective case-control	Hematological diseases	143	Median (Range): 41 (18–69)	Proven/probable vs. non-IA	Serum	EORTC/MSG-2008
Prattes 2015 [47]	Austria	Prospective cohort	Hematological diseases	76	Median (IQR): 61 (52–69)	Probable vs. non-IA	BALF	EORTC/MSG-2008
Miceli 2015 [48]	USA	Prospective cohort	Hematological diseases	314	Mean (Range): 61 (35–81)	Proven/probable vs. non-IA	BALF	EORTC/MSG-2008
Johnson 2015 [49]	UK	Retrospective case-control	Immunocompromised patients	23	≥ 18	Proven vs. non-IA	BALF	EORTC/MSG-2008
Eigl 2015 [50]	Austria	Prospective cohort	Hematologic malignancies	128	Median (Range): 60 (19–85)	Proven/probable vs. non-IA	BALF	EORTC/MSG-2008
Willinger 2014 [51]	Austria	Prospective cohort	Transplant patients	36	Median (Range): 51 (18–71)	Proven/probable vs. non-IA	BALF	EORTC/MSG-2008
Prattes 2014 [52]	Austria	Retrospective case-control	Respiratory diseases	196	Median (Range): 64 (18–92)	Proven/probable vs. non-IA	BALF	EORTC/MSG-2008
Hoenigl 2014 [53]	Austria	Retrospective case-control	Immunocompromised patients	51	Median (Range): 58 (24–77)	Proven/probable vs. non-IA	BALF	EORTC/MSG-2008
White 2013 [54]	UK	Retrospective case-control	Hematological diseases	81	Mean: 48.3	Proven/probable vs. non-IA	Serum	EORTC/MSG-2008
Held 2013 [55]	Germany	Prospective cohort	Transplant patients	101	NR	Proven/probable vs. non-IA	Serum	EORTC/MSG-2008

Note: IA: Invasive *aspergillosis*; IQR: Interquartile Range; BALF: Bronchoalveolar lavage fluid; EORTC/MSG: European Organization of Research and Treatment of Cancer/Mycoses Study Group; ECMM/ISHAM: European Confederation of Medical Mycology/International Society for Human and Animal Mycology

### Results of diagnostic meta-analysis

#### Results of LFD diagnosis

**Fig. 3:**
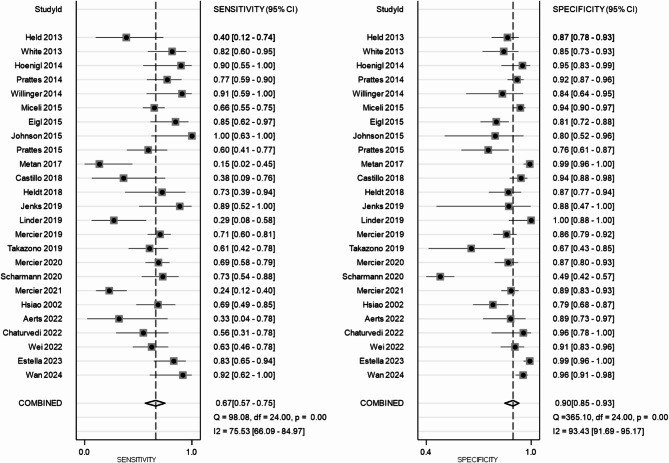
Forest plot of pooled sensitivity and specificity of lateral flow device (LFD)

**Fig. 4:**
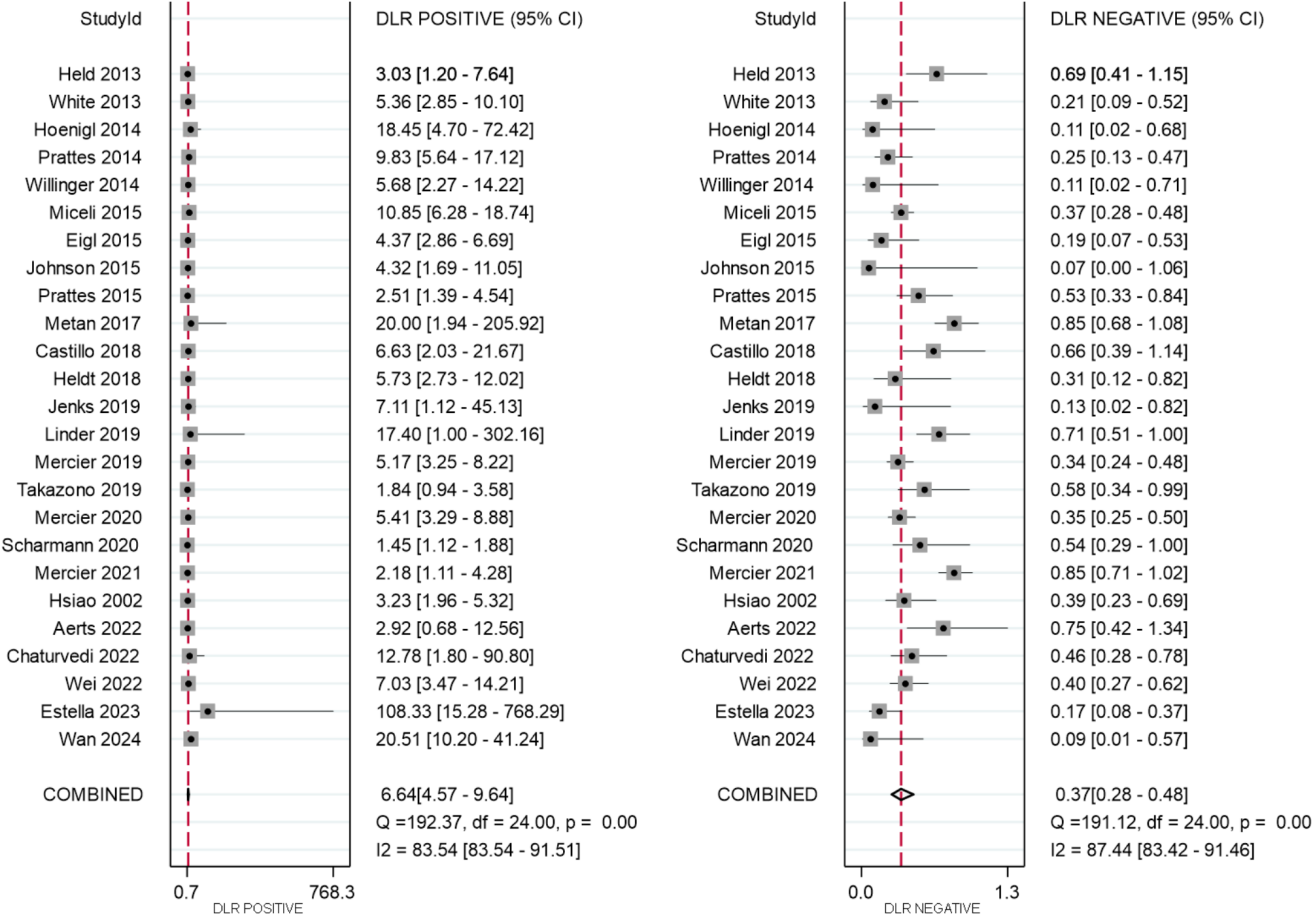
Forest plot of pooled positive likelihood ratio (PLR) and negative likelihood ratio (NLR) of lateral flow device (LFD)

**Fig. 5:**
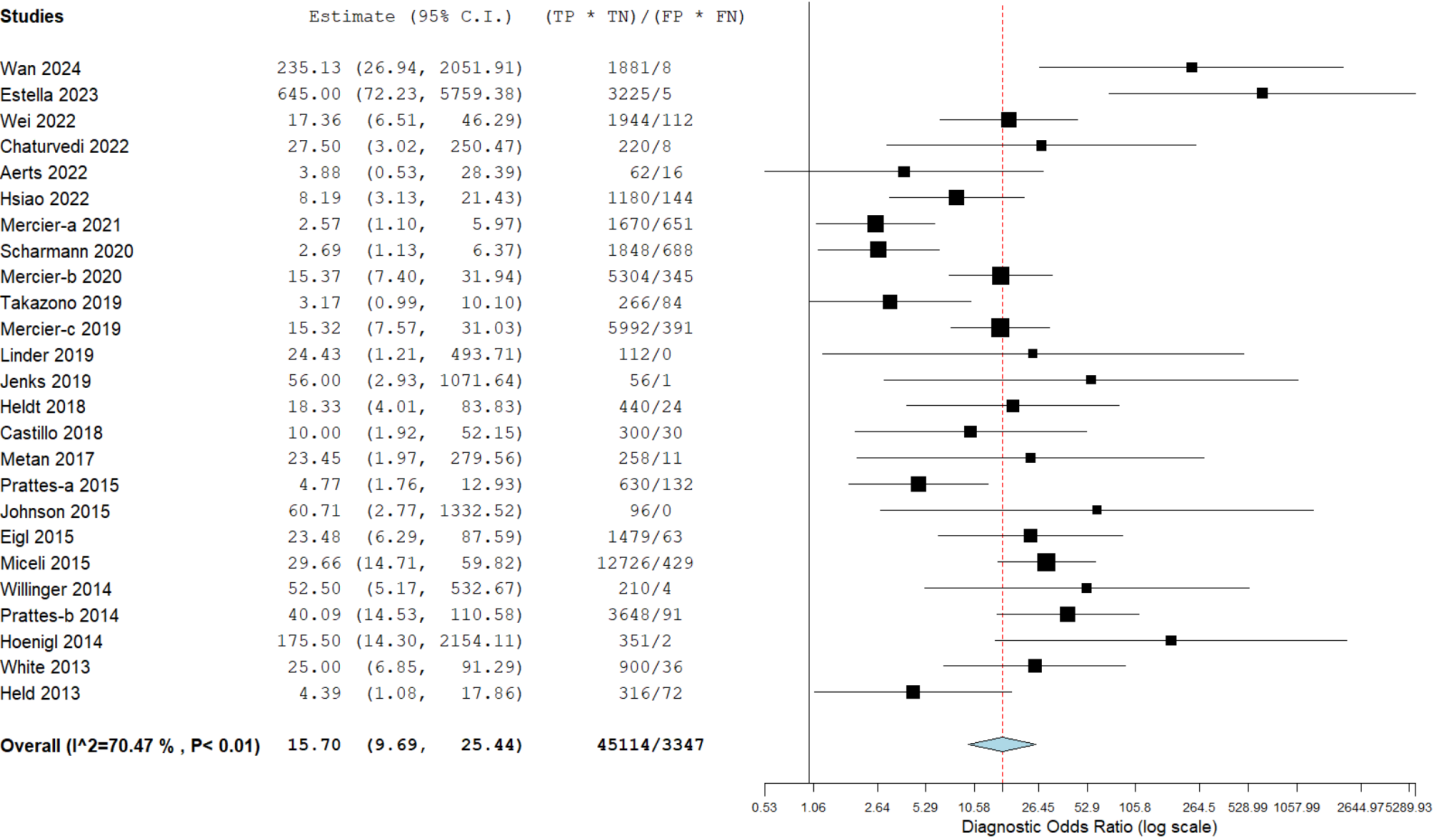
Forest plot of diagnostic odds ratio (DOR) of lateral flow device (LFD)

**Fig. 6:**
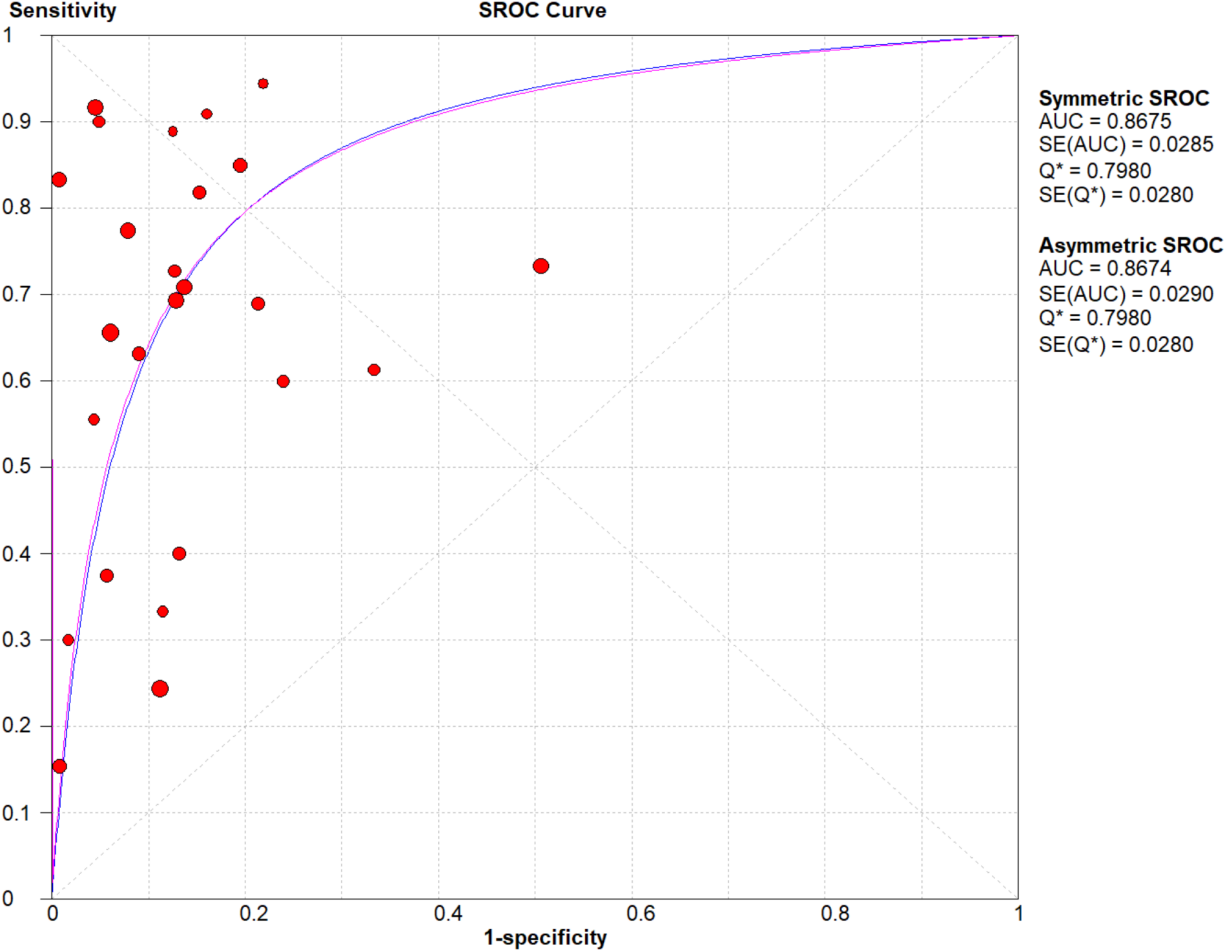
Summary receiver operating characteristic curves (SROC) plots of sensitivity and specificity

All 25 included studies provided complete diagnostic performance data from LFD testing for both proven/probable IA cases and non-IA controls. The pooled sensitivity and pooled specificity were 0.67 (95% CI: 0.57–0.75) and 0.90 (95% CI: 0.85–0.93), respectively (Fig. 3). The pooled PLR and NLR were 6.64 (95% CI: 4.57–9.64) and 0.37 (95% CI: 0.28–0.48), respectively (Fig. 4), and DOR was 15.70 (95% CI: 9.69–25.44) (Fig. 5). The SROC curve is displayed in Fig. 6 and represents the relationship between sensitivity and specificity throughout the study, and the area under the SROC curve (AUC) was 0.87, thereby indicating that the LFD had a credible diagnostic capability.

#### Results of subgroup analysis

The subgroup analysis was performed based on four factors (Table 2). The results revealed that among the specimen sources, BALF was reported in 16 studies while serum was reported in 10 studies. Notably, there was a substantial disparity in sensitivity between these two sources (BALF: 0.72 vs. serum: 0.49), although no significant difference was observed in their final diagnostic accuracy (AUROC: BALF − 0.89 vs. serum − 0.80). In the case definition, three forms were included, proven/probable vs. non-IA, proven vs. non-IA, and probable vs. non-IA, which were reported in 18, 6, and 3 studies, respectively. The results of the meta-analysis demonstrated that among the three forms, LFD exhibited the highest sensitivity (0.75, 95% CI: 0.66–0.82) and specificity (0.90, 95% CI: 0.87–0.93) for diagnosing “proven vs. non-IA”, with an AUROC value of 0.86 obtained; meanwhile, a higher AUROC value of 0.88 was observed in “proven/probable vs. non-IA”. In the “probable vs. non-IA” classification, the test demonstrated a sensitivity of 0.66 (95% CI: 0.56–0.75) and specificity of 0.85 (95% CI: 0.80–0.89), with the AUROC value reaching 0.52. Among study designs, the case-control study was superior to the cohort study in sensitivity (0.70 vs. 0.61), while displaying lower specificity (0.85 vs. 0.89); however, there was no significant disparity in diagnostic accuracy between these two study designs (both AUROC, 0.86). Finally, among the population characteristics, the results of the meta-analysis showed that the highest sensitivity (0.94) was generated in population immunocompromised patients, followed by population with respiratory diseases (0.71). In transplant patients, LFD yielded the lowest sensitivity (0.49) but high specificity (0.91). The population with hematological diseases included the largest number of studies (k = 14), and the results showed that the sensitivity of LFD was 0.64, the specificity was 0.89, and the AUROC value was 0.88. Further details are provided in appendix figure S2.1-S12.5.

**Table 2:** Results of subgroup analysis of the four factors

Subgroup	No. of studies	Pooled Sensitivity	Pooled Specificity	Pooled PLR	Pooled NLR	Diagnostic OR	AUROC (SE)
**Specimen Source**							
BALF	16	0.72 (0.67, 0.76)	0.85 (0.83, 0.87)	6.04 (3.85, 9.49)	0.35 (0.26, 0.46)	22.36 (12.25, 40.81)	0.89 (0.028)
Serum	10	0.49 (0.41, 0.56)	0.91 (0.89, 0.93)	4.37 (2.56, 7.47)	0.61 (0.46, 0.81)	8.76 (4.00, 19.15)	0.80 (0.077)
**Definition**							
Proven/probable vs. non-IA	18	0.65 (0.60, 0.56)	0.87 (0.86, 0.89)	5.90 (3.77, 9.24)	0.42 (0.31, 0.58)	16.07 (9.30, 27.77)	0.88 (0.029)
Proven vs. non-IA	6	0.75 (0.66, 0.82)	0.90 (0.87, 0.93)	6.54 (2.98, 14.39)	0.29 (0.16, 0.51)	31.66 (7.20, 139.22)	0.86 (0.065)
Probable vs. non-IA	3	0.66 (0.56, 0.75)	0.85 (0.80, 0.89)	4.18 (2.50, 6.98)	0.42 (0.32, 0.55)	10.15 (4.63, 22.26)	0.52 (0.515)
**Study design**							
Retrospective case-control	13	0.70 (0.65, 0.75)	0.85 (0.83, 0.87)	6.12 (3.40, 10.99)	0.37 (0.25, 0.58)	18.59 (9.37, 36.89)	0.86 (0.034)
Prospective cohort	12	0.61 (0.56, 0.66)	0.89 (0.87, 0.91)	4.90 (3.24, 7.40)	0.45 (0.31, 0.64)	13.51 (6.55, 27.88)	0.86 (0.054)
**Population**							
Hematological diseases	14	0.64 (0.59, 0.68)	0.89 (0.88, 0.91)	5.32 (3.81, 7.44)	0.41 (0.29, 0.59)	14.35 (8.37, 24.85)	0.88 (0.029)
Respiratory diseases	5	0.71 (0.64, 0.78)	0.80 (0.76, 0.83)	5.91 (1.75, 20.03)	0.37 (0.24, 0.57)	17.09 (3.86, 75.63)	0.76 (0.063)
Transplant patients	4	0.49 (0.33, 0.65)	0.91 (0.87, 0.94)	4.88 (2.79, 8.53)	0.64 (0.44, 0.94)	10.87 (3.70, 31.93)	0.89 (0.077)
Immunocompromised patients	2	0.94 (0.73, 1.00)	0.91 (0.80, 0.97)	8.19 (1.96, 34.26)	0.09 (0.02, 0.43)	115.12 (16.43, 806.49)	—

Note: Data format: Effect (95% confidence interval); OR: Odds Ratio; IA: Invasive aspergillosis; BALF: Bronchoalveolar lavage fluid; PLR: Positive Likelihood Ratio; NLR: Negative Likelihood Ratio; AUROC: Area under the Receiver-Operating Characteristic curve

### Certainty of evidence

Certainty of evidence summary can be seen in Table 3. In the initial setting, the sensitivity was 0.64 (95% CI: 0.53–0.73), and the specificity was 0.89 (95% CI: 0.86–0.92). In addition, the pre-test probability was set at 20% based on the results of the SROC. Regarding the sensitivity outcome, all 25 studies provided results pertaining to the diagnosis of LFD. When evaluating the overall quality of evidence, key factors contributing to downgrading included studies with a high risk of bias, substantial statistical heterogeneity, and imprecision (indicated by wide confidence intervals), ultimately resulting in a very low quality of evidence. As for specificity, again 25 studies were included and, unlike sensitivity, this measure was not downgraded due to imprecision. Neither index was downgraded due to publication bias, as the funnel plot and quantitative test did not detect the presence of publication bias (*P* = 0.68, supplementary figure S13). Finally, the level of evidence for sensitivity tests was very low, whereas specificity was low.

**Table 3:** Summary of evidence quality for the diagnosis of IA by LFD

Outcome	№ of studies (№ of patients)	Study design	Factors that may decrease certainty of evidence					Effect per 1,000 patients tested	Test accuracy CoE
			Risk of bias	Indirectness	Inconsistency	Imprecision	Publication bias	pre-test probability of 20%	
**True positives**	25 studies 682 patients	cohort & case-control type studies	serious^a^	not serious	serious^b^	serious^c^	not serious	128 (106 to 146)	⨁◯◯◯ Very low^a, b,c^
**False negatives**								72 (54 to 94)	
**True negatives**	25 studies 2251 patients	cohort & case-control type studies	serious^a^	not serious	serious^b^	not serious	not serious	712 (688 to 736)	⨁⨁◯◯ Low^a, b^
**False positives**								88 (64 to 112)	

**Annotation:** True positives - patients with [target condition]; False negatives - patients incorrectly classified as not having [target condition]; True negatives - patients without [target condition]; False positives - patients incorrectly classified as having [target condition].

a. High risk of bias.

b. High statistical heterogeneity.

c. Too wide a confidence interval.

## Discussion

The findings from this meta-analysis revealed that the pooled sensitivity of LFD for diagnosing IA was 0.67 (95% CI: 0.57–0.75), while the pooled specificity was 0.90 (95% CI: 0.85–0.93). These results indicate that LFD exhibit a diagnostic accuracy of approximately 67% in identifying patients with proven/probable IA, compared to an accuracy rate of 90% in non-IA patients, the results of sensitivity and specificity showed that LFD was more accurate in diagnosing non-IA patients than proven/probable IA patients. The true positive rate of LFD in the IA population was 15 times higher than that in the control population, as determined by DOR. Additionally, the NLR values indicated that the LFD technique had a 6.6-fold greater likelihood of detecting IA in patients compared to controls, providing strong diagnostic evidence but not reaching convincing levels (PLR > 10). Overall, considering all indicators, the AUC accuracy for LFD reached 86%, demonstrating its value for diagnosing IA.

Histopathological demonstration of tissue invasion by *Aspergillus* hyphae remains the gold standard for proven IA, as per the 2020 EORTC/MSG consensus criteria [27, 56]. However, the applicability of this diagnostic method is limited in certain scenarios, such as critically ill patients or those with thrombocytopenia, thereby necessitating the advancement of alternative diagnostic approaches. While our analysis confirmed the value of LFDs in IA diagnosis, their clinical significance requires a comparative assessment of their accuracy against other established diagnostic methods. A 2023 meta-analysis [57] directly compared serum Galactomannan and β-D-glucan assays, reporting a sensitivity/specificity of 0.54/0.94 for Galactomannan versus 0.72/0.82 for β-D-glucan. Our findings demonstrated that the sensitivity of LFD (BALF or serum) falls between that of Galactomannan and β-D-glucan, whereas there was no significant difference in terms of specificity among these three techniques. However, the apparent specificity advantage of β-D-glucan (0.82) requires cautious interpretation, as this assay may yield false-positive results in patients with Candida/Pneumocystis infections, thereby overestimating its specificity for IA [58]. In contrast, LFD demonstrates stable specificity across clinical scenarios due to its *Aspergillus*-specific epitope recognition, making it a preferable rule-out tool in populations with high fungal co-infection prevalence [59]. In another systematic review [60], the authors reported on the diagnostic accuracy of PCR for probable IA in immunocompromised patients. The results demonstrated that serum PCR exhibited a sensitivity of 0.81 and a specificity of 0.79 for diagnosing IA. These findings suggest that while PCR has a high true positive rate for IA diagnosis, its diagnostic accuracy is significantly reduced in non-IA populations. Conversely, our study revealed that LFD had a higher specificity than PCR (BALF/serum: 0.90; serum alone: 0.91), suggesting its superior diagnostic accuracy in non-infected populations. A 2022 overview [61] indicated that serum PCR demonstrated sensitivity and specificity of 0.84–0.88 and 0.75–0.76, respectively, while BALF PCR showed relatively reduced sensitivity (0.77–0.80) but higher specificity (0.94–0.95) compared to serum PCR. Notably, pediatric populations exhibited lower PCR accuracy (sensitivity: 0.82; specificity: 0.73). Our subgroup analysis revealed comparable diagnostic performance of LFD in BALF (sensitivity: 0.72; specificity: 0.85) without showing significant advantages over BALF PCR. These findings offer valuable insights for future investigations of LFD. Subsequent studies should not only explore how different sample sources affect the diagnostic accuracy of LFD, but also examine the impact of age variations (particularly between adults and children) on its diagnostic performance. A critical methodological concern arises from the common practice of defining IA cases by Galactomannan positivity via enzyme-linked immunosorbent assay (ELISA) in studies lacking histopathological confirmation. This approach may introduce incorporation bias, as the polyclonal antibodies utilized in certain LFD - such as the JF5 monoclonal antibody targeting extracellular glycoproteins-exhibit epitope homology with β-1,5-galactofuranoside residues detected by Galactomannan assays [34]. Empirical evidence suggests that this antigenic cross-reactivity (attributable to shared glycan epitopes or polysaccharide moieties) could artificially inflate LFD sensitivity and specificity estimates when Galactomannan-positive criteria are used for case definition [46]. To mitigate this bias, future research should prioritize the inclusion of histologically confirmed IA cases or establish clear distinctions between Galactomannan-dependent and Galactomannan-independent subgroups. Additionally, the complementary diagnostic value of LFD in combination with other diagnostic modalities warrants further investigation to enhance the accuracy and reliability of IA diagnosis.

The clinical applicability of LFD for IA diagnosis warrants cautious interpretation due to heterogeneous factors, including specimen source variability (e.g., BALF vs. serum), study design inconsistencies, and divergent patient population characteristics, which collectively resulted in an overall evidence certainty downgrade-with very low certainty for sensitivity estimates and low certainty for specificity estimates as per GEADE criteria. To elucidate the influence of various factors on diagnostic accuracy, we performed a subgroup analysis based on study design (case-control vs. cohort), sample source (BALF vs. serum), case definition (proven/probable IA vs. non-IA), and population characteristics (diseases). However, due to limited number of available studies, we were unable to quantitatively explore the impact of sample size and gold standard on evidence quality. The meta-analysis revealed high statistical heterogeneity in the pooled sensitivity and specificity estimates, leading to a downgrade in evidence quality due to this heterogeneity. The cause of such statistical heterogeneity may be attributed to inconsistencies between studies. Furthermore, imprecision (evidenced by wide 95% confidence intervals for specificity estimates) was observed, resulting in a degradation of evidence quality. Insufficient sample sizes in certain studies, particularly those involving proven/probable IA cases with limited samples - likely contributed to this imprecision. To address these issues, future studies should adopt standardized diagnostic criteria and implement LFDs using unified protocols (e.g., standardized sample collection timing, interpretation criteria, and quality control) to enhance consistency. Comparability across study groups should be ensured through stratification or matching of participants by age, immune status, and comorbidities, while exploring the impact of patient characteristics on LFD performance. Large-scale studies featuring adequate sample sizes, prospective designs, and diverse populations, supported by comprehensive data collection, remain essential to verify the robustness and accuracy of the findings.

It should be acknowledged that in the practical application of utilizing LFD for diagnosing IA, the amalgamation of patients’ clinical characteristics may contribute more effectively to the accurate identification of infection. For instance, a study [62] demonstrated a potential association between patient age and IA infection, highlighting its role as an independent risk factor. Additionally, neutropenia, HIV/AIDS, kidney transplant recipients, and renal failure have been identified as key predisposing conditions for IA [9]. Considering the disease characteristics of the population included in this meta-analysis, it is suggested that immunocompromised patients exhibit an elevated risk of IA. To optimize LFD performance, clinicians should systematically account for these risk factors during diagnostic workflows. Subgroup analyses revealed significant heterogeneity in the diagnostic performance of LFD across sample types. Specifically, the sensitivity the sensitivity of LFD in BALF samples (0.72) was markedly higher than in serum samples (0.49), suggesting that BALF is a superior specimen for IA diagnosis in patients with high clinical suspicion of infection. These results are consistent with a systematic review, which also reported better diagnostic accuracy for BALF-derived galactomannan compared to serum-based assays [63]. Notably, the observed sensitivity advantage of BALF testing (0.72 vs. 0.49) with comparable overall accuracy (AUROC 0.89 vs. 0.80; Δ = 0.09, *P* = 0.072) likely reflects precision trade-offs, BALF’s slightly lower specificity (0.85 vs. 0.91) counterbalanced its sensitivity gains. The borderline significance (*P* = 0.072) may indicate limited statistical power (68%) due to serum subgroup’s smaller sample size (*n* = 1,057 vs. BALF *n* = 2,014), suggesting potential Type II error. While BALF-based LFD testing demonstrates enhanced diagnostic sensitivity relative to serum-based methods, its clinical application (BALF specimen collection) is constrained by two principal limitations. First, the invasive bronchoscopy procedure carries inherent risks of respiratory complications and hemorrhagic events, particularly in immunocompromised patient populations [64]. Second, the methodology necessitates specialized infrastructure for standardized specimen processing and temperature-controlled transportation protocols to ensure diagnostic reliability [14]. A 2019 systematic review evaluating the combined use of Galactomannan and LFD for IA diagnosis [65], The authors reported a sensitivity of 0.93 and a specificity of 0.82, demonstrating that LFD with GM assays significantly enhances the accuracy of IA diagnosis. Overall, the findings of LFD alone or in combination with other techniques for diagnosing IA provide implications for future research. First, it is recommended to conduct more high-quality studies focusing on LFD-based diagnostics. Second, active exploration should be undertaken to investigate the diagnostic efficacy of combining LFD with other diagnostic technologies for IA, aiming to identify the optimal combined diagnostic approach. In resource-limited settings, LFDs emerge as a critical diagnostic tool for IA due to their rapid, cost-effective, and user-friendly characteristics [34]. These devices empower clinicians to facilitate early intervention, optimize therapeutic decision-making, and enhance healthcare accessibility, particularly for populations in primary care settings and high-risk patient cohorts. Further validation of their diagnostic performance and technological refinements are warranted to expand their global applicability.

Our study has certain limitations. Firstly, only English studies were considered during the literature acquisition process, and no studies in other languages were included. This linguistic bias may disproportionately exclude evidence from regions with distinct healthcare ecosystems. The exclusion of such regional data could artificially inflate diagnostic accuracy estimates, as LFD performance characteristics are known to vary with local prevalence of antigenic cross-reactants and immunosuppression etiologies. Secondly, while subgroup analysis was conducted to analyze differences for four variables in the heterogeneity analysis of this study, quantitative analysis could not be performed for other factors due to a limited number of studies available. Consequently, only qualitative interpretation was provided regarding the sources of heterogeneity.

## Conclusions

The findings of this diagnostic meta-analysis indicate that LFD is a valuable diagnostic modality for IA. While both BALF and serum are viable specimens for LFD testing, BALF-based assays achieved significantly higher sensitivity, strongly supporting their prioritization in clinical practice.

## Electronic supplementary material

Below is the link to the electronic supplementary material.

Supplementary Material 1

## Data Availability

The data that support the findings of this study are available from the corresponding author upon reasonable request.

## Abbreviations

IA: Invasive aspergillosis
LFD: Lateral flow device
CI: Confidence intervals
SROC: Summary receiver operating characteristic curves
AUC: Area Under the Curve
CAPA: COVID-19-associated pulmonary aspergillosis
PCR: Polymerase chain reaction
BDG: β-D-glucan
BALF: Bronchoalveolar lavage fluid
EORTC/MSG: European Organization for Research and Treatment of Cancer/Mycoses Study Group
QUADAS: Quality assessment of diagnostic accuracy studies
STARD: Standards for reporting diagnostic accuracy
GRADE: Grades of Recommendation, Assessment, Development and Evaluation
PLR: Positive likelihood ratios
NLR: Negative likelihood ratios
DOR: Diagnostic odds ratio
ELISA: Enzyme-linked immunosorbent assay
