# Supplementary material for: Lateral-flow device for the diagnosis of invasive aspergillosis: a systematic review and diagnostic meta-analysis
Source: BMC Infect Dis. 2025 Mar 20;25:388. doi: 10.1186/s12879-025-10769-x (PMC11924699; doi:10.1186/s12879-025-10769-x)
Supplement: Supplementary file 1 — Supplementary Material 1 [file 12879_2025_10769_MOESM1_ESM.docx]

**Table 1 PRISMA-DTA Checklist Item**

| **Section/topic** | **#** | **PRISMA-DTA Checklist Item** | **Reported on page #** |
| --- | --- | --- | --- |
| **TITLE / ABSTRACT** | | | 2 |
| Title | 1 | Identify the report as a systematic review (+/- meta-analysis) of diagnostic test accuracy (DTA) studies. | 2 |
| Abstract | 2 | Abstract: See PRISMA-DTA for abstracts. | 2 |
| **INTRODUCTION** | | | 3-5 |
| Rationale | 3 | Describe the rationale for the review in the context of what is already known. | 3 |
| Clinical role of index test | D1 | State the scientific and clinical background, including the intended use and clinical role of the index test, and if applicable, the rationale for minimally acceptable test accuracy (or minimum difference in accuracy for comparative design). | 4 |
| Objectives | 4 | Provide an explicit statement of question(s) being addressed in terms of participants, index test(s), and target condition(s). | 5 |
| **METHODS** | | | 5-8 |
| Protocol and registration | 5 | Indicate if a review protocol exists, if and where it can be accessed (e.g., Web address), and, if available, provide registration information including registration number. | NA |
| Eligibility criteria | 6 | Specify study characteristics (participants, setting, index test(s), reference standard(s), target condition(s), and study design) and report characteristics (e.g., years considered, language, publication status) used as criteria for eligibility, giving rationale. | 5-6 |
| Information sources | 7 | Describe all information sources (e.g., databases with dates of coverage, contact with study authors to identify additional studies) in the search and date last searched. | 5 |
| Search | 8 | Present full search strategies for all electronic databases and other sources searched, including any limits used, such that they could be repeated. | 5 |
| Study selection | 9 | State the process for selecting studies (i.e., screening, eligibility, included in systematic review, and, if applicable, included in the meta-analysis). | 6 |
| Data collection process | 10 | Describe method of data extraction from reports (e.g., piloted forms, independently, in duplicate) and any processes for obtaining and confirming data from investigators. | 6 |
| Definitions for data extraction | 11 | Provide definitions used in data extraction and classifications of target condition(s), index test(s), reference standard(s) and other characteristics (e.g. study design, clinical setting). | 6 |
| Risk of bias and applicability | 12 | Describe methods used for assessing risk of bias in individual studies and concerns regarding the applicability to the review question. | 6 |
| Diagnostic accuracy measures | 13 | State the principal diagnostic accuracy measure(s) reported (e.g. sensitivity, specificity) and state the unit of assessment (e.g. per-patient, per-lesion). | 7-8 |
| Synthesis of results | 14 | Describe methods of handling data, combining results of studies and describing variability between studies. This could include, but is not limited to: a) handling of multiple definitions of target condition. b) handling of multiple thresholds of test positivity, c) handling multiple index test readers, d) handling of indeterminate test results, e) grouping and comparing tests, f) handling of different reference standards | 7-8 |
| Meta-analysis | D2 | Report the statistical methods used for meta-analyses, if performed. | 7-8 |
| Additional analyses | 16 | Describe methods of additional analyses (e.g., sensitivity or subgroup analyses, meta-regression), if done, indicating which were pre-specified. | 8 |
| **RESULTS** | | | 8-10 |
| Study selection | 17 | Provide numbers of studies screened, assessed for eligibility, included in the review (and included in meta-analysis, if applicable) with reasons for exclusions at each stage, ideally with a flow diagram. | 8 |
| Study characteristics | 18 | For each included study provide citations and present key characteristics including: a) participant characteristics (presentation, prior testing), b) clinical setting, c) study design, d) target condition definition, e) index test, f) reference standard, g) sample size, h) funding sources | 8 |
| Risk of bias and applicability | 19 | Present evaluation of risk of bias and concerns regarding applicability for each study. | 8-9 |
| Results of individual studies | 20 | For each analysis in each study (e.g. unique combination of index test, reference standard, and positivity threshold) report 2x2 data (TP, FP, FN, TN) with estimates of diagnostic accuracy and confidence intervals, ideally with a forest or receiver operator characteristic (ROC) plot. | 8-9 |
| Synthesis of results | 21 | Describe test accuracy, including variability; if meta-analysis was done, include results and confidence intervals. | 9 |
| Additional analysis | 23 | Give results of additional analyses, if done (e.g., sensitivity or subgroup analyses, meta-regression; analysis of index test: failure rates, proportion of inconclusive results, adverse events). | 9-10 |
| **DISCUSSION** | | | 11-16 |
| Summary of evidence | 24 | Summarize the main findings including the strength of evidence. | 11-16 |
| Limitations | 25 | Discuss limitations from included studies (e.g. risk of bias and concerns regarding applicability) and from the review process (e.g. incomplete retrieval of identified research). | 16 |
| Conclusions | 26 | Provide a general interpretation of the results in the context of other evidence. Discuss implications for future research and clinical practice (e.g. the intended use and clinical role of the index test). | 16 |
| **FUNDING** | | |  |
| Funding | 27 | For the systematic review, describe the sources of funding and other support and the role of the funders. | Title Page |

*Adapted From:*  McInnes MDF, Moher D, Thombs BD, McGrath TA, Bossuyt PM, The PRISMA-DTA Group (2018). Preferred Reporting Items for a Systematic Review and Meta-analysis of Diagnostic Test Accuracy Studies: The PRISMA-DTA Statement. JAMA. 2018 Jan 23;319(4):388-396. doi: 10.1001/jama.2017.19163.

**Supplementary analyses for the diagnostic meta-analysis**


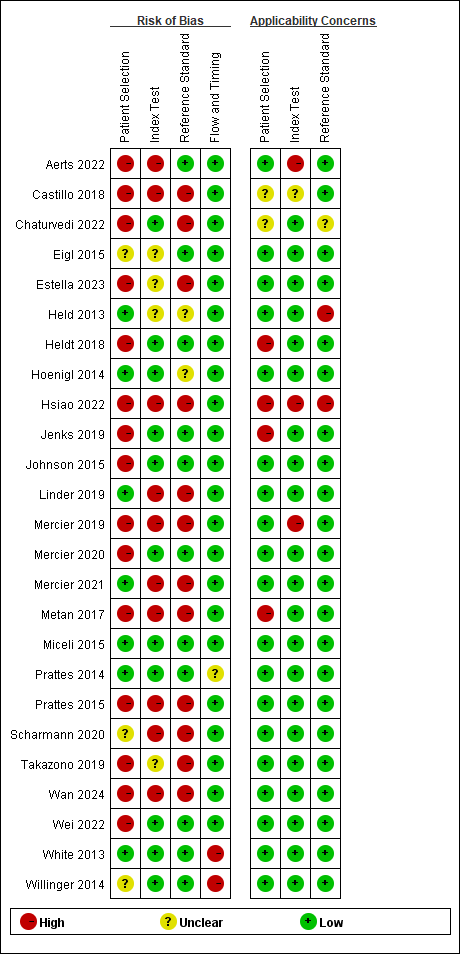


Figure S1. The assessment results for specific items (domains) of each study

Figure S2.1-S2.6 Results of BALF subgroup analysis


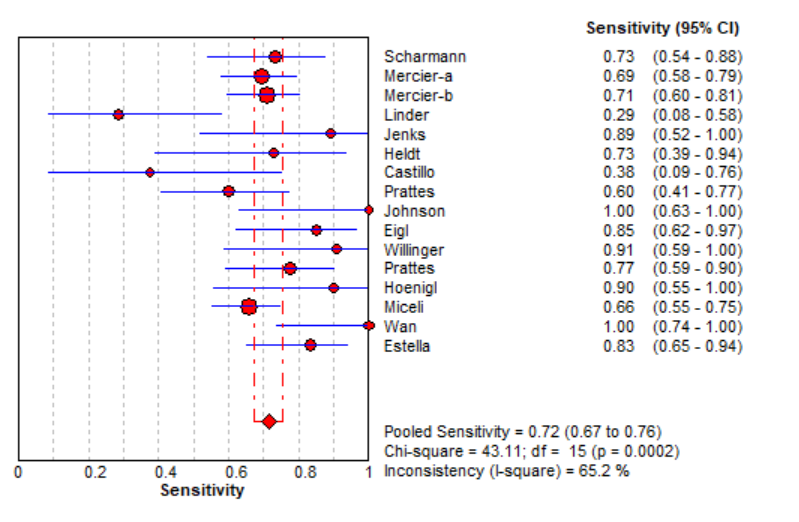


Figure S2.1 Sensitivity of BALF subgroup analysis


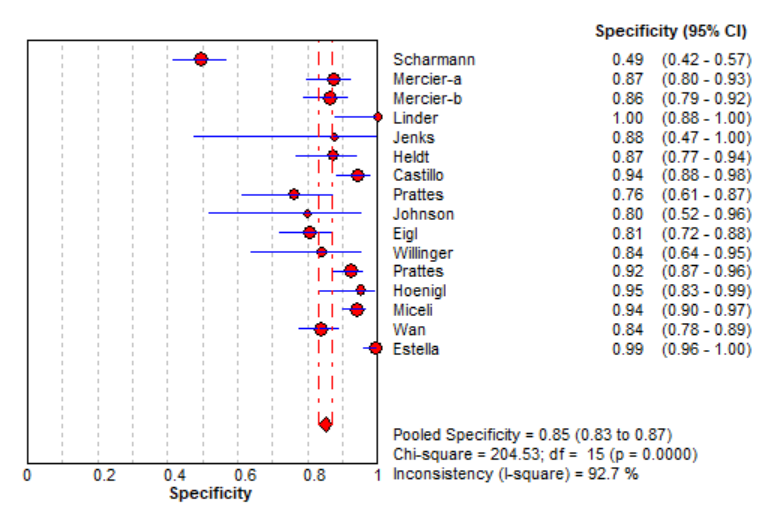


Figure S2.2 Specificity of BALF subgroup analysis


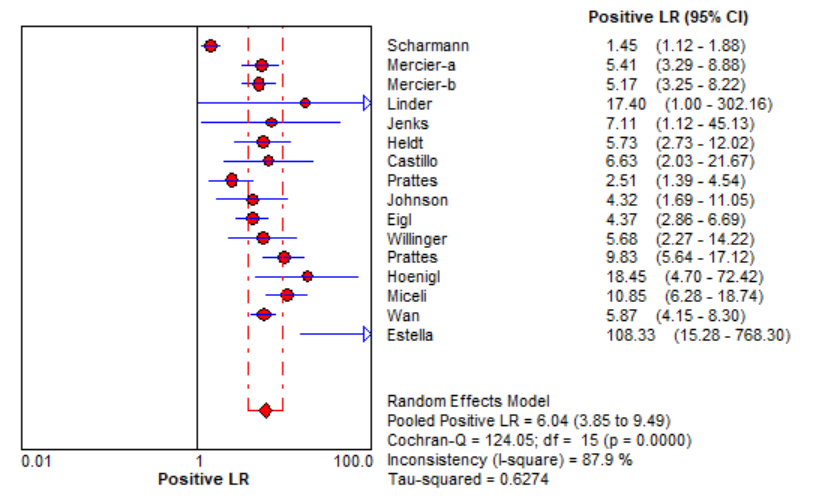


Figure S2.3 PLR of BALF subgroup analysis


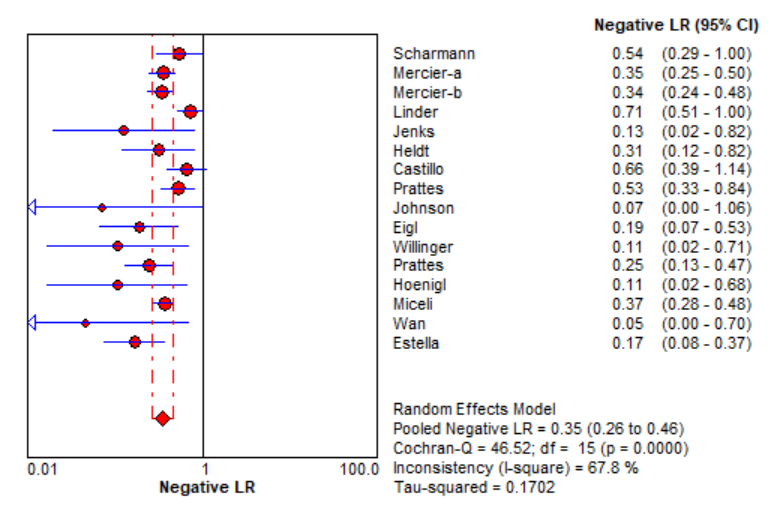


Figure S2.4 NLR of BALF subgroup analysis

Diagnostic OR


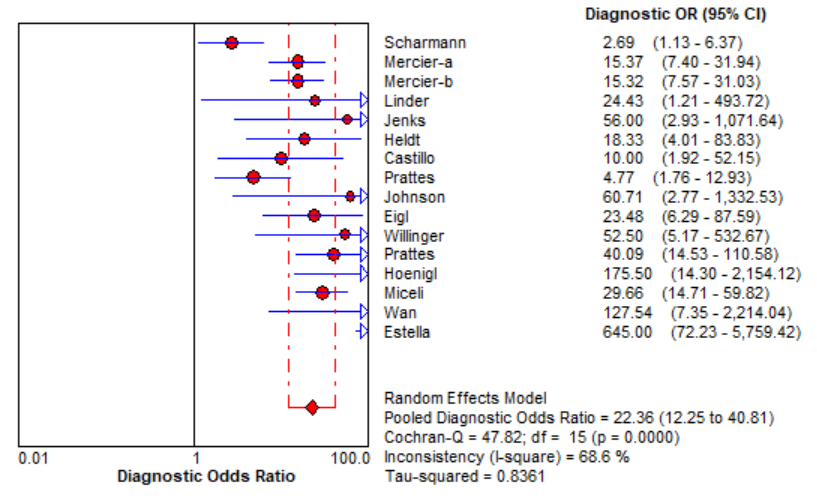


Figure S2.5 DOR of BALF subgroup analysis


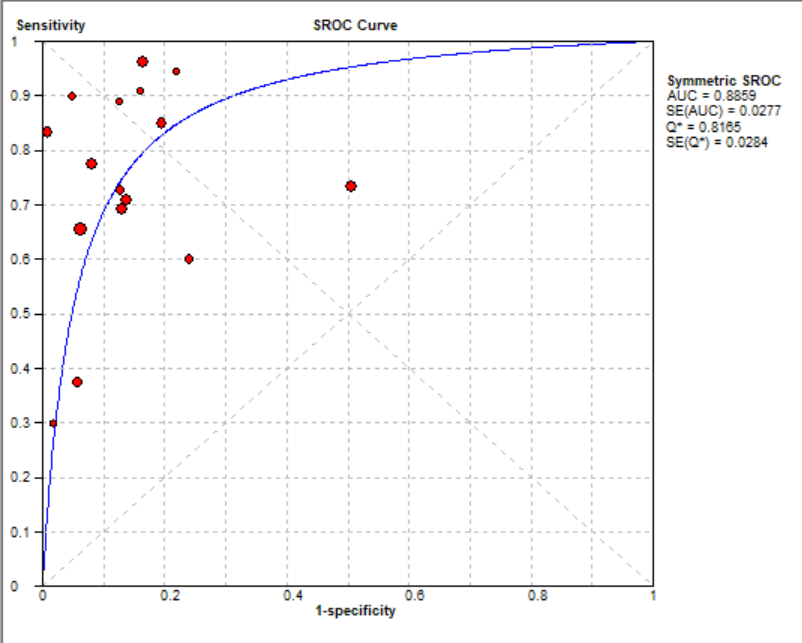


Figure 2.6 AUROC of BALF subgroup analysis

**Figure S3.1-S3.6 Results of Serum subgroup analysis**


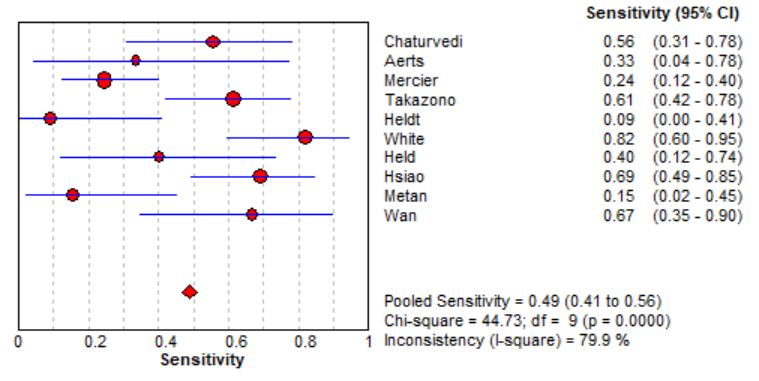


Figure S3.1 Sensitivity of Serum subgroup analysis


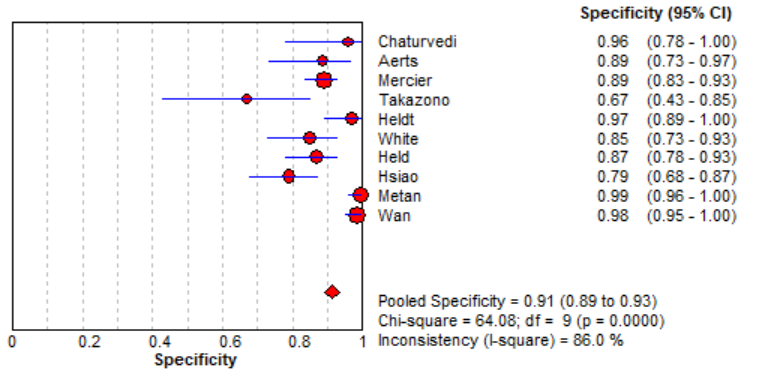


Figure S3.2 Specificity of Serum subgroup analysis


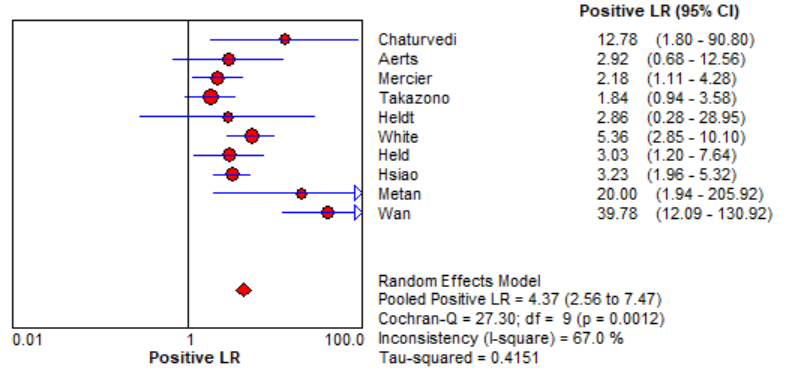


Figure S3.3 PLR of Serum subgroup analysis


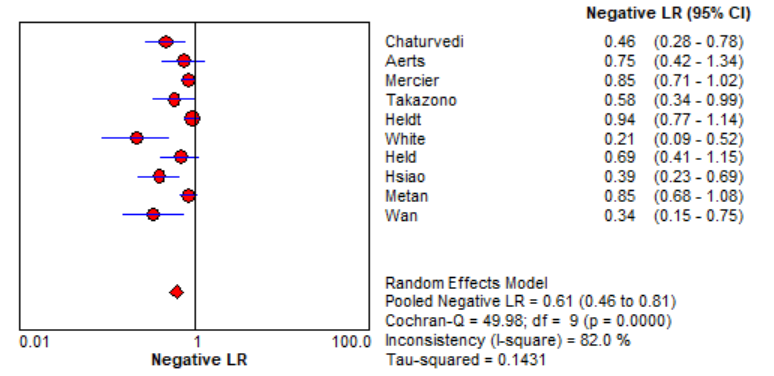


Figure S3.4 NLR of Serum subgroup analysis


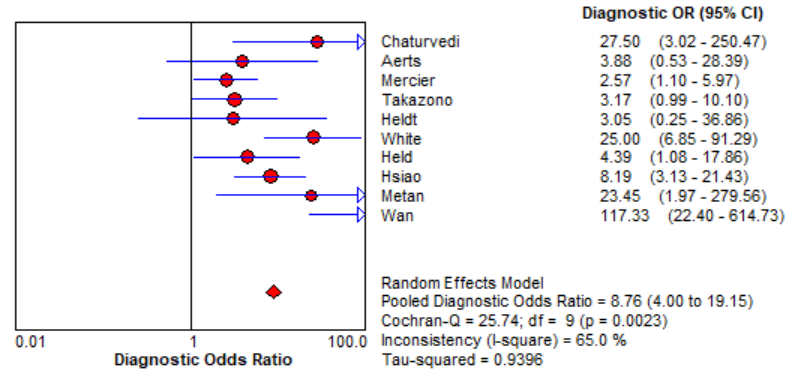


Figure S3.5 DOR of Serum subgroup analysis


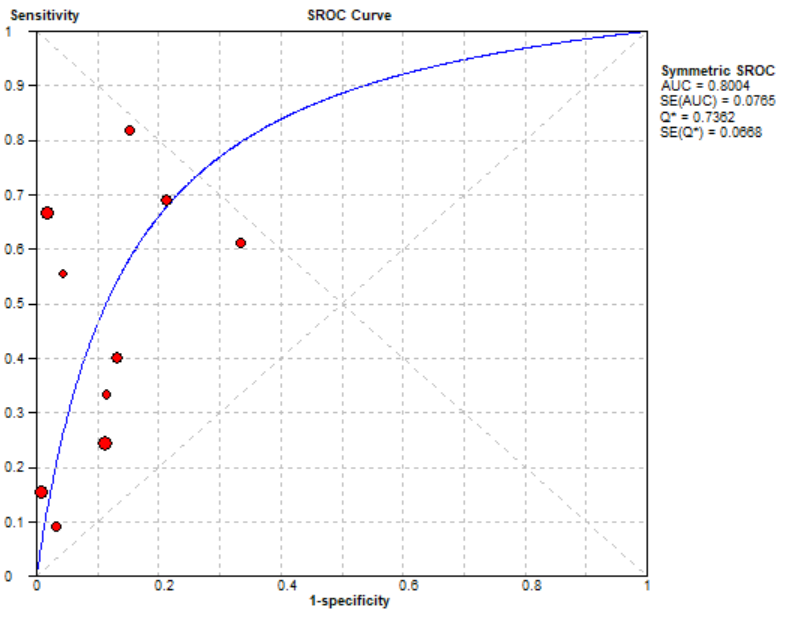


Figure S3.6 AUSROC of Serum subgroup analysis

**Figure S4.1-4.6 Results of Proven/probable vs. non-IA subgroup analysis**


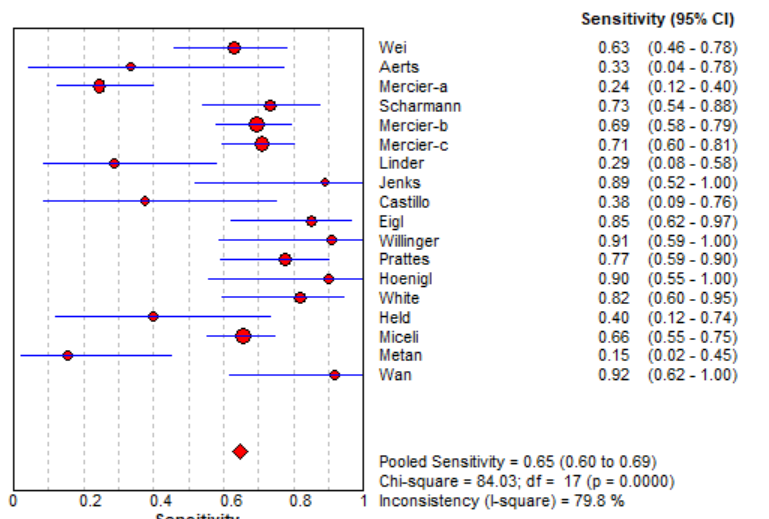


Figure S4.1 Sensitivity of Proven/probable vs. non-IA subgroup analysis


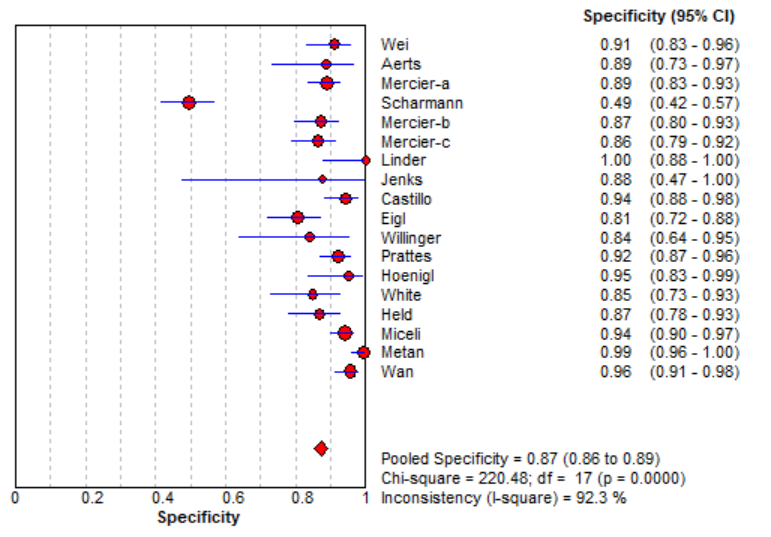


Figure S4.2 Specificity of Proven/probable vs. non-IA subgroup analysis


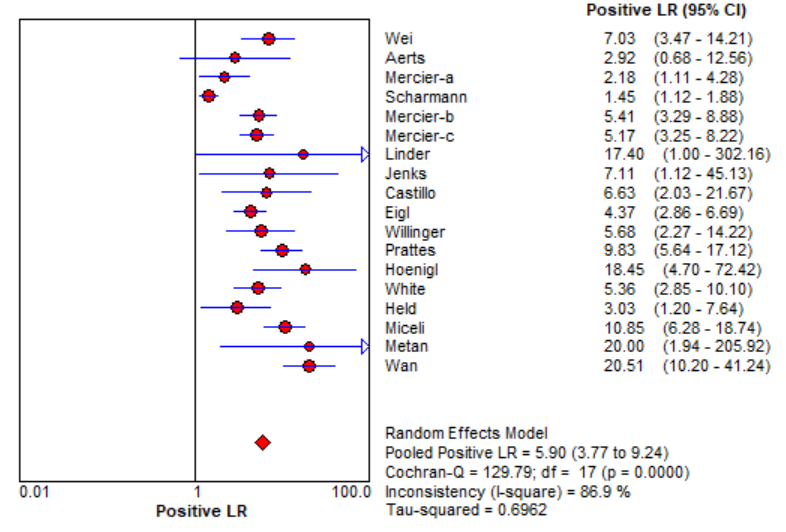


Figure S4.3 PLR of Proven/probable vs. non-IA subgroup analysis


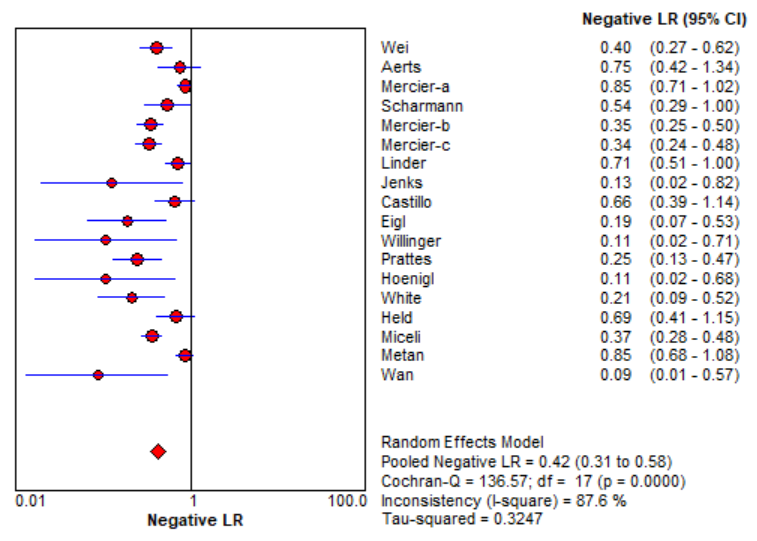


Figure S4.4 NLR of Proven/probable vs. non-IA subgroup analysis


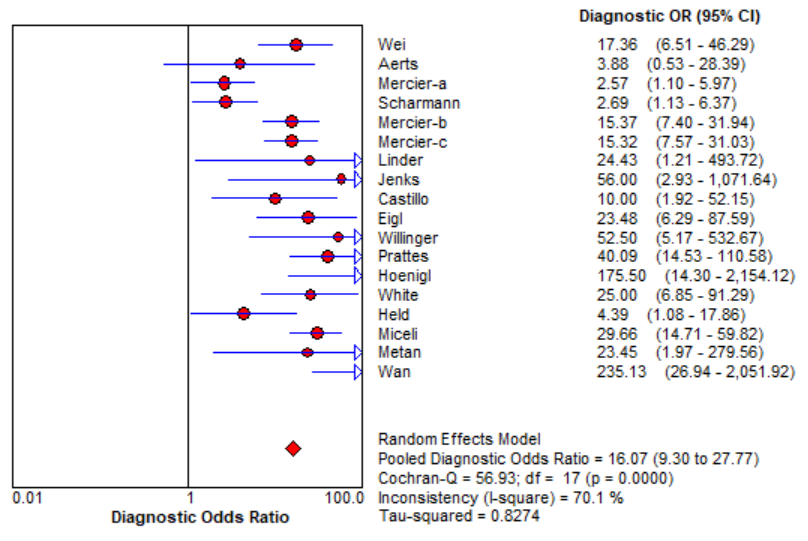


Figure S4.5 DOR of Proven/probable vs. non-IA subgroup analysis


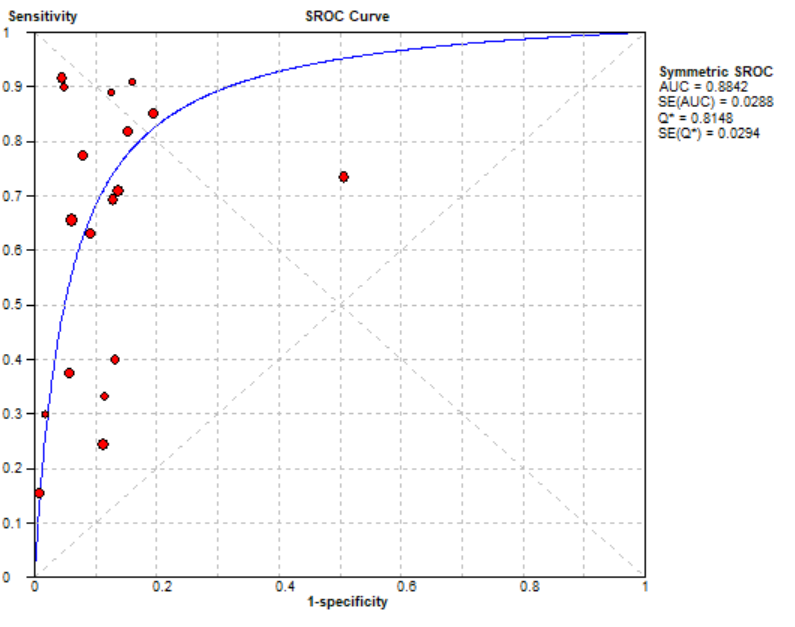


Figure S4.6 AUCSROC of Proven/probable vs. non-IA subgroup analysis

**Figure S5.1-5.6 Results of** **Proven vs. non-IA subgroup analysis**


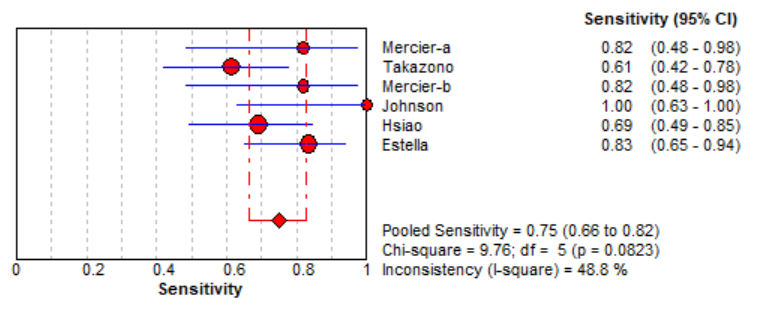


Figure S5.1 Sensitivity of Proven vs. non-IA subgroup analysis


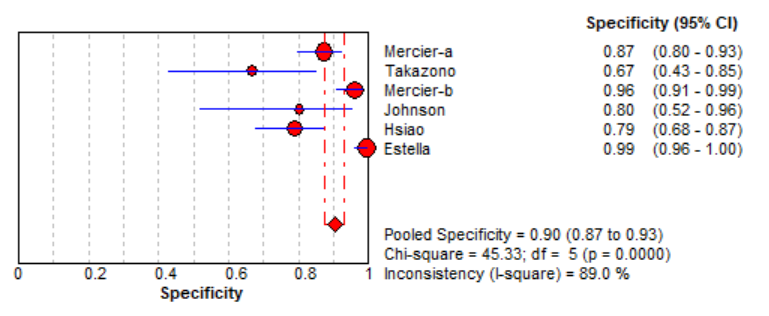


Figure S5.2 Specificity of Proven vs. non-IA subgroup analysis


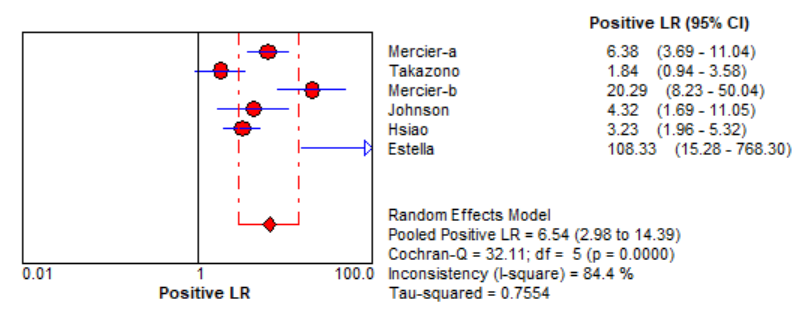


Figure S5.3 PLR of Proven vs. non-IA subgroup analysis


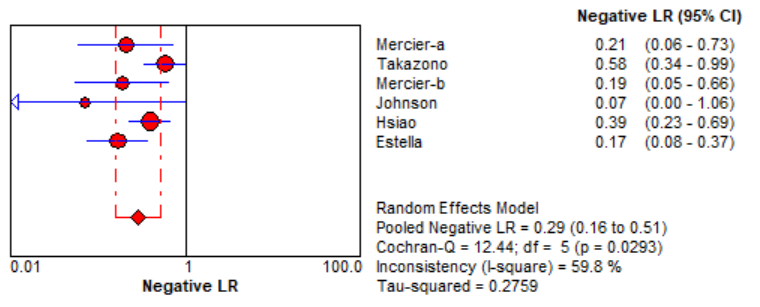


Figure S5.4 NLR of Proven vs. non-IA subgroup analysis


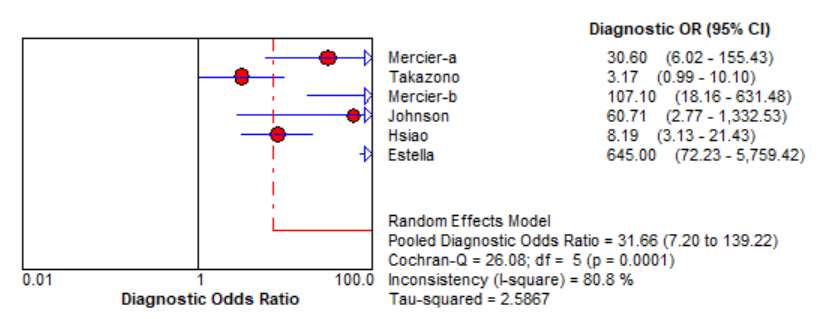


Figure S5.5 DOR of Proven vs. non-IA subgroup analysis


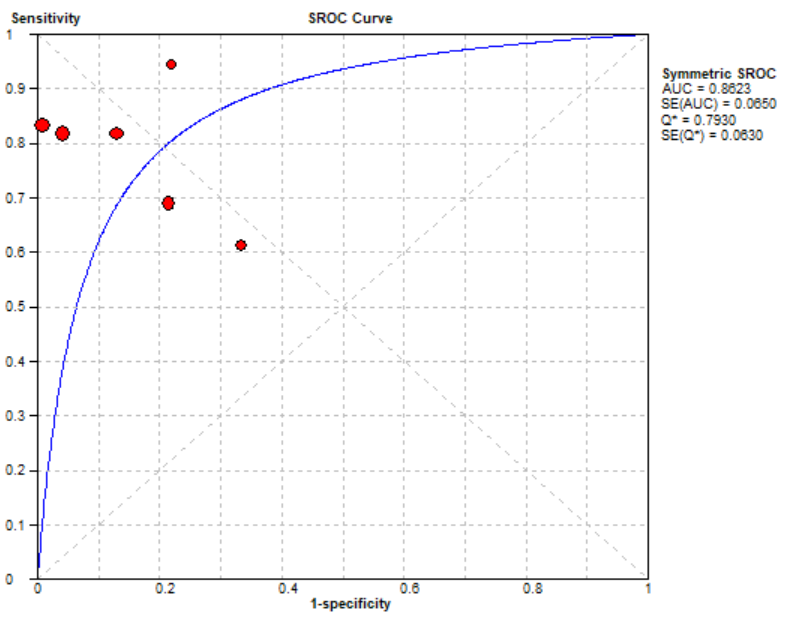


Figure S5.6 AUROC of Proven vs. non-IA subgroup analysis

**Figure S6.1-6.6 Results of** **Probable vs. non-IA subgroup analysis**


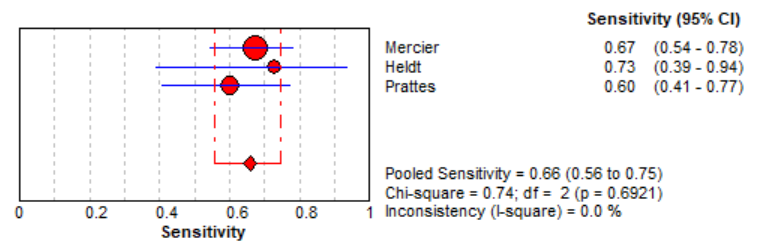


Figure S6.1 Sensitivity of Probable vs. non-IA subgroup analysis


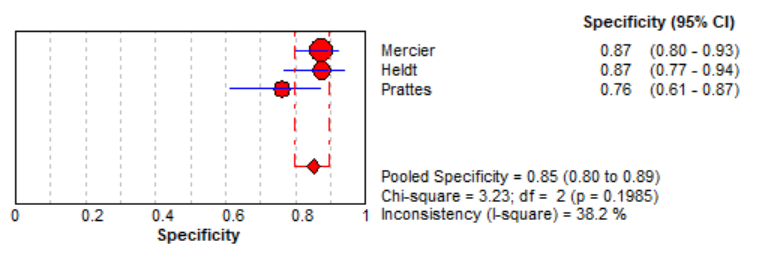


Figure S6.2 Specificity of Probable vs. non-IA subgroup analysis


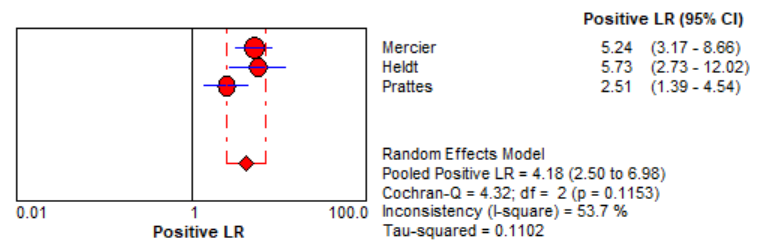


Figure S6.3 PLR of Probable vs. non-IA subgroup analysis


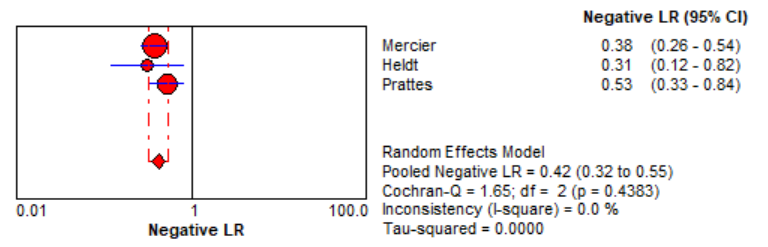


Figure S6.4 NLR of Probable vs. non-IA subgroup analysis


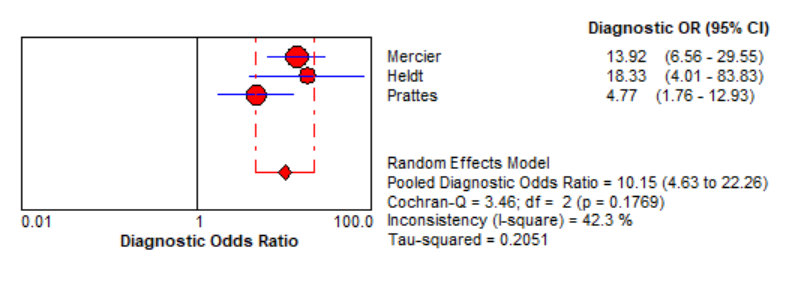


Figure S6.5 DOR of Probable vs. non-IA subgroup analysis


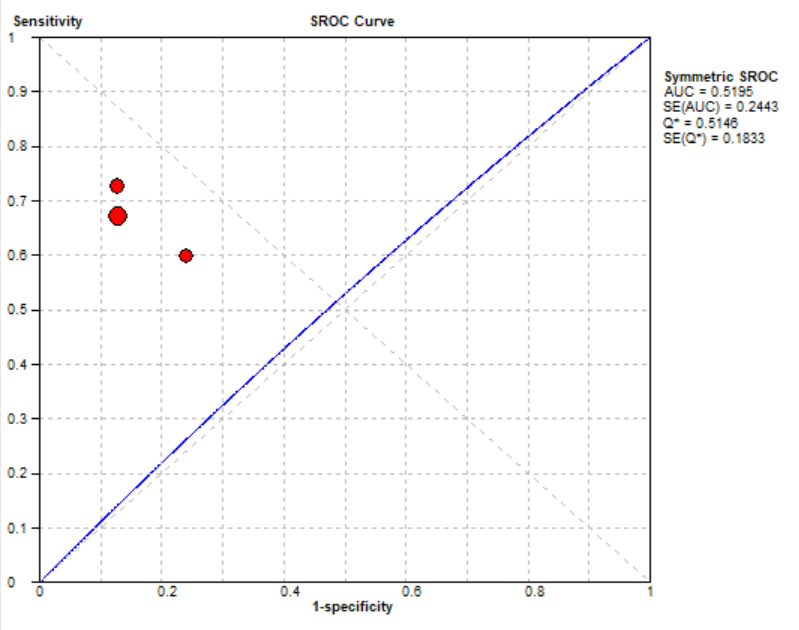


Figure S6.6 AUROC of Probable vs. non-IA subgroup analysis

**Figure S7.1-7.6 Results of** **Retrospective case-control subgroup analysis**


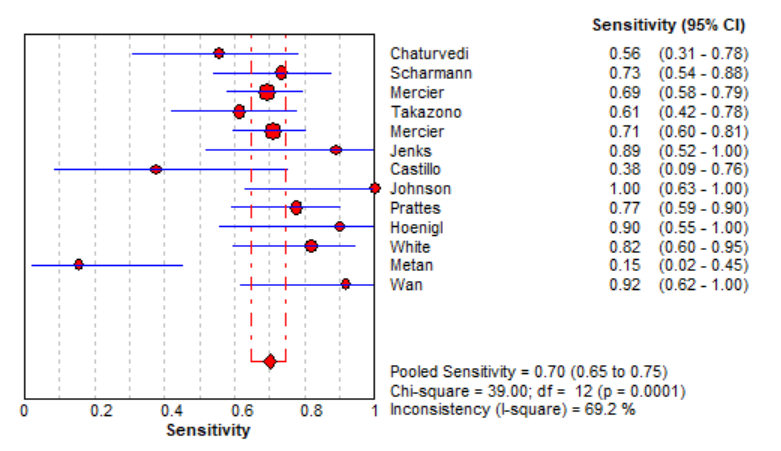


Figure S7.1 Sensitivity of Retrospective case-control subgroup analysis


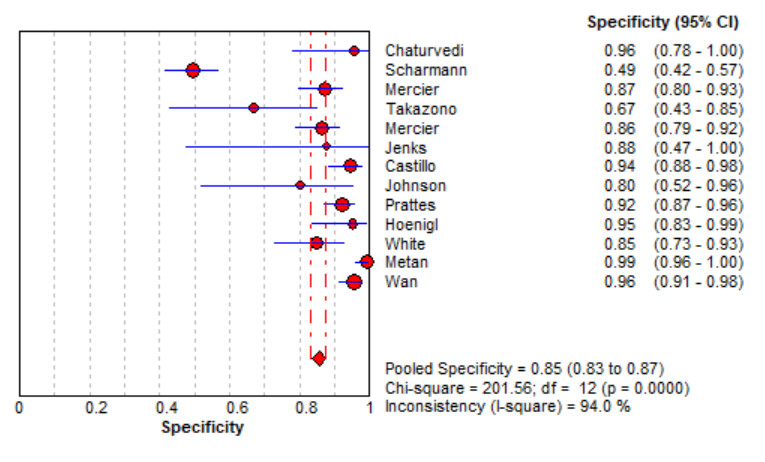


Figure S7.2 Specificity of Retrospective case-control subgroup analysis


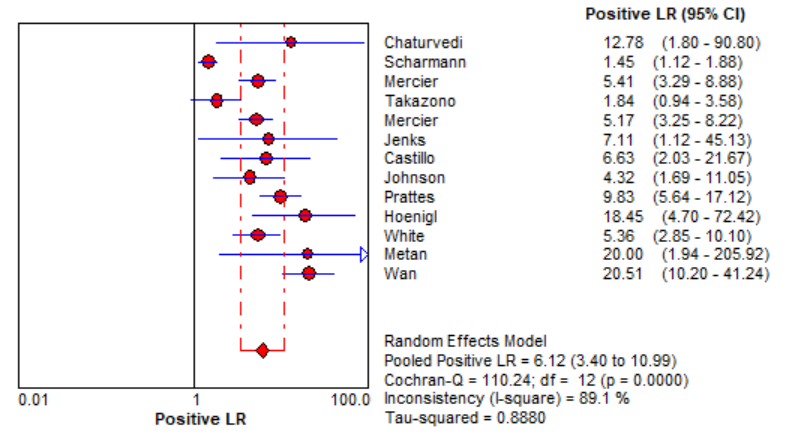


Figure S7.3 PLR of Retrospective case-control subgroup analysis


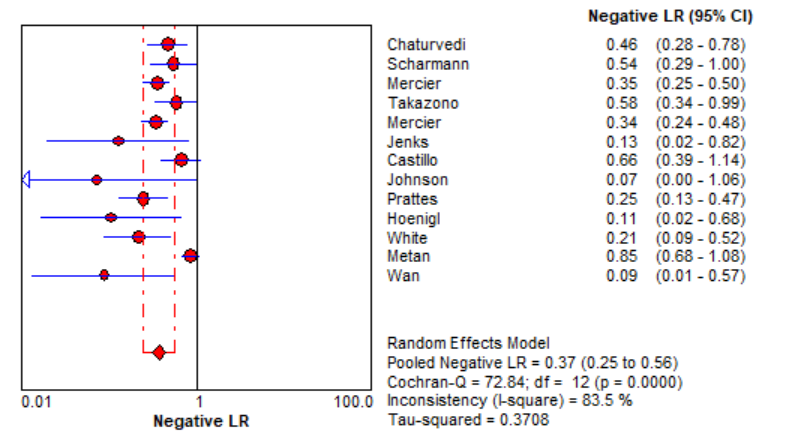


Figure S7.4 NLR of Retrospective case-control subgroup analysis


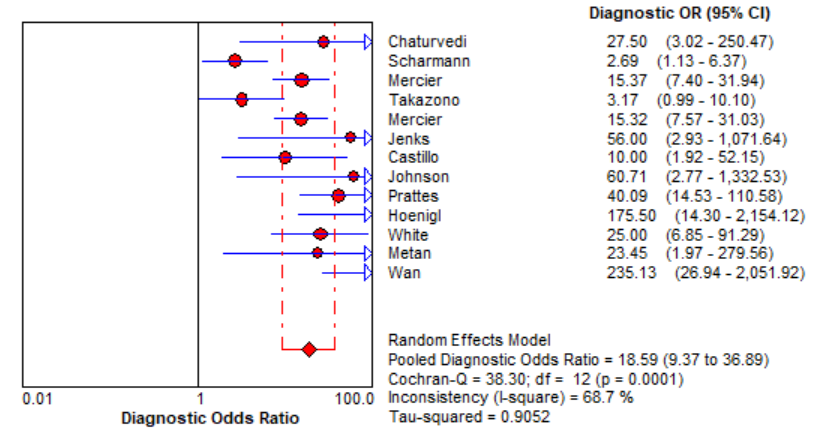


Figure S7.5 DOR of Retrospective case-control subgroup analysis


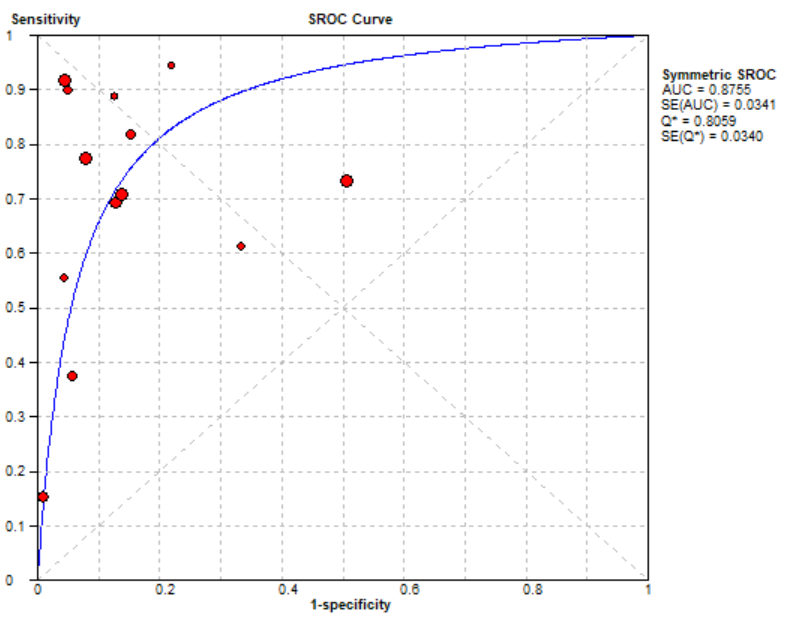


Figure S7.6 AUROC of Retrospective case-control subgroup analysis

**Figure S8.1-8.6 Results of** **Prospective cohort subgroup analysis**

Pooled


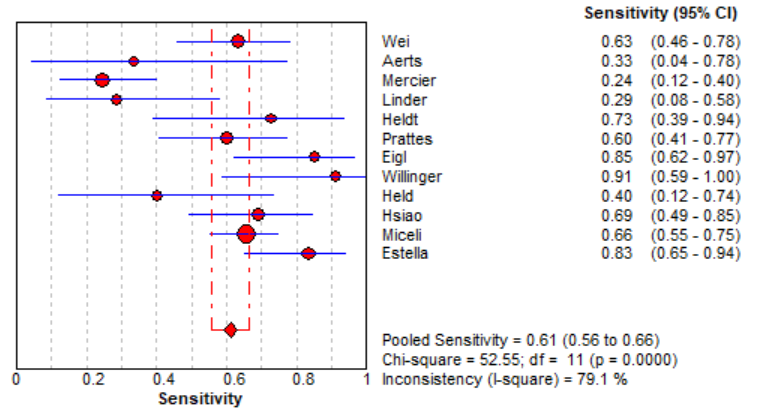


Figure S8.1 Sensitivity of Prospective cohort subgroup analysis


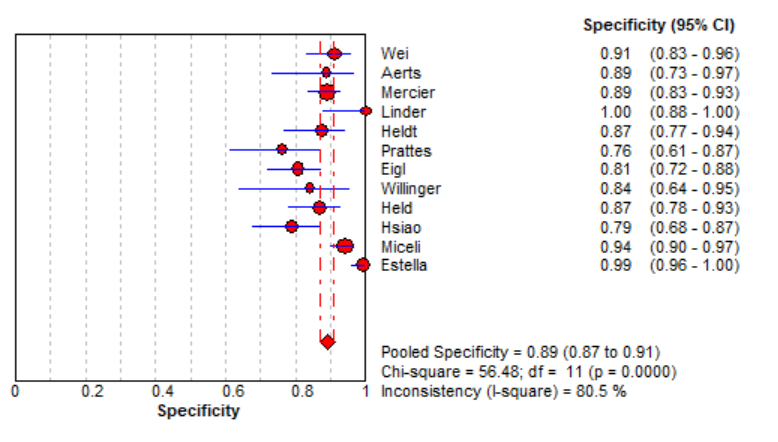


Figure S8.2 Specificity of Prospective cohort subgroup analysis


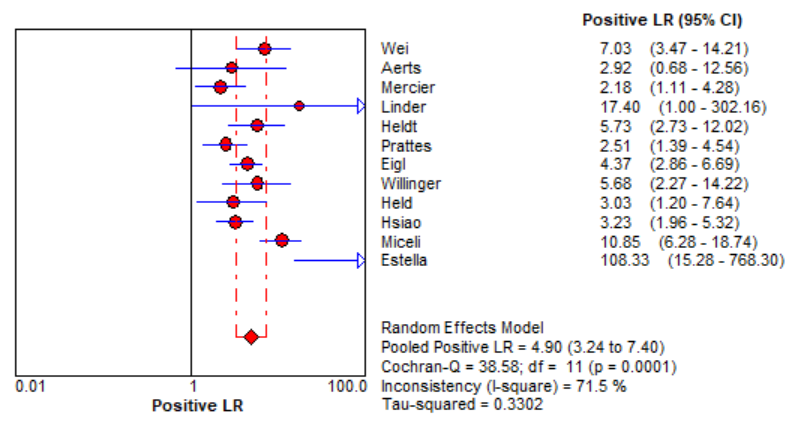


Figure S8.3 PLR of Prospective cohort subgroup analysis


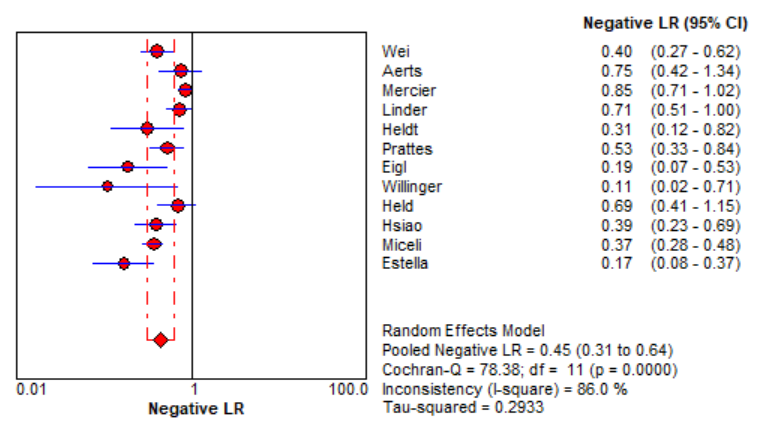


Figure S8.4 NLR of Prospective cohort subgroup analysis


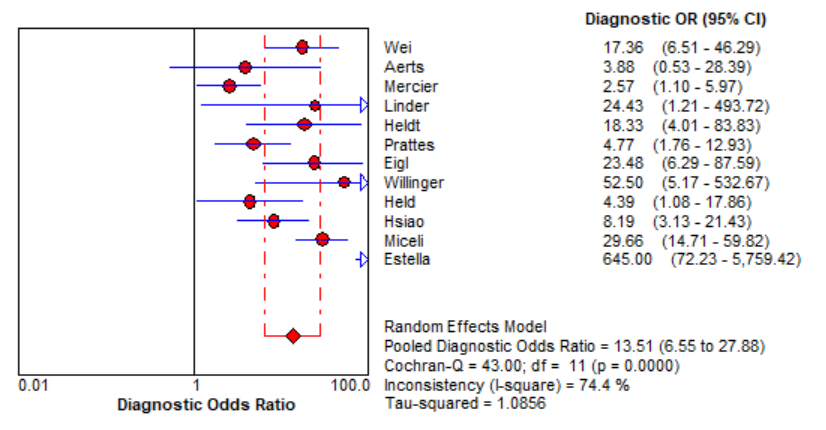


Figure S8.5 DOR of Prospective cohort subgroup analysis


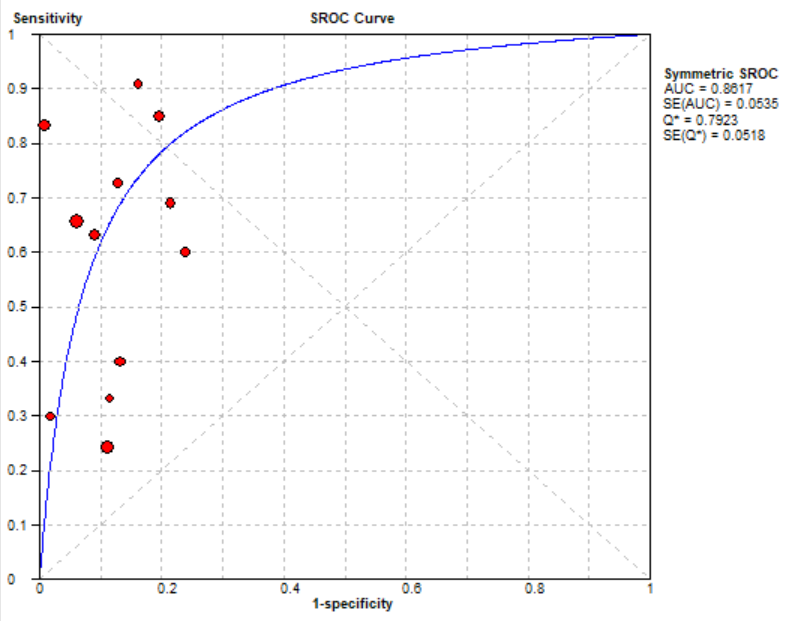


Figure S8.6 AUROC of Prospective cohort subgroup analysis

**Figure S9.1-9.6 Results of** **Hematological diseases subgroup analysis**


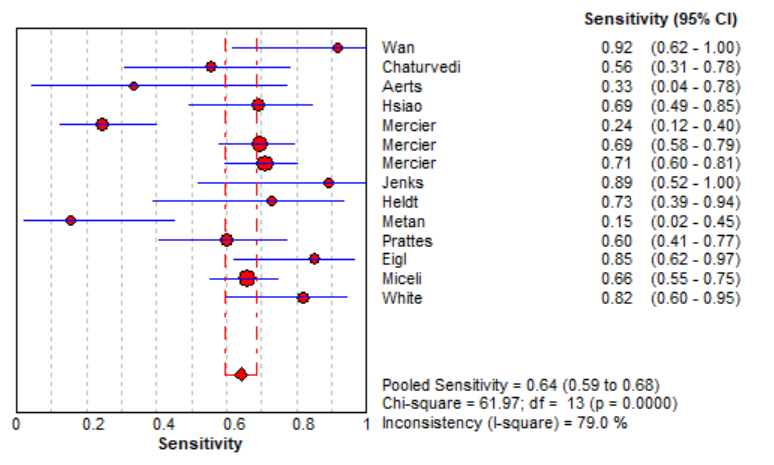


Figure S9.1 Sensitivity of Hematological diseases subgroup analysis


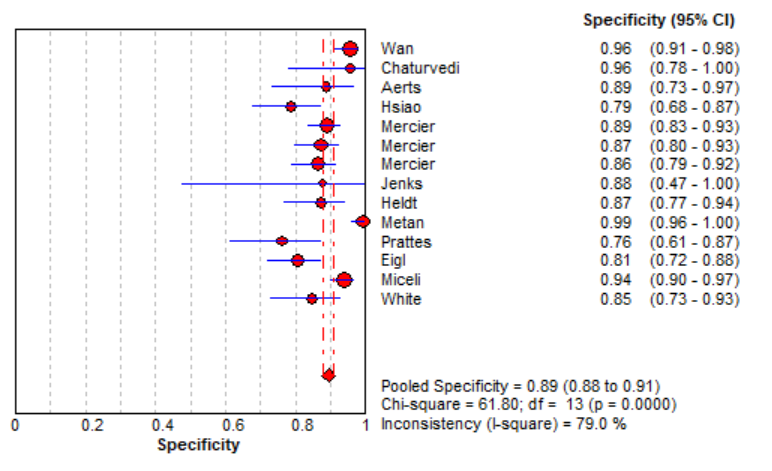


Figure S9.2 Specificity of Hematological diseases subgroup analysis


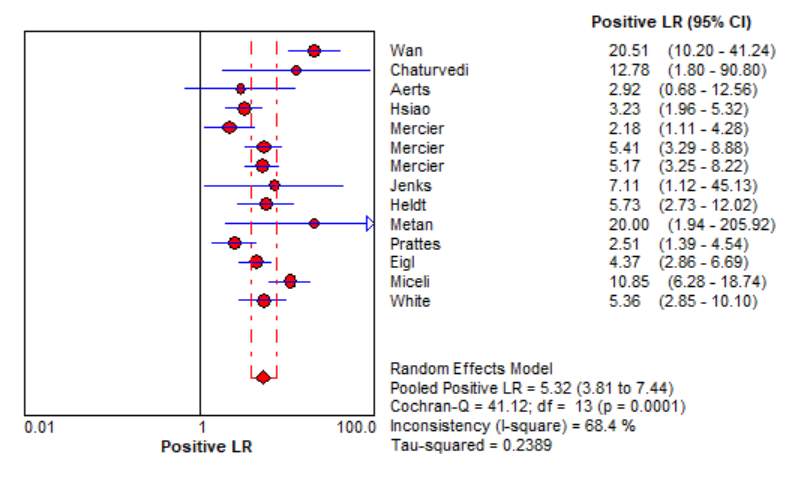


Figure S9.3 PLR of Hematological diseases subgroup analysis


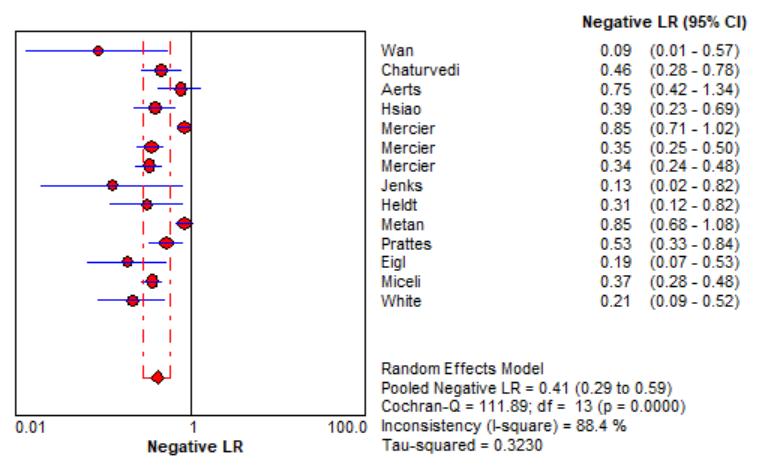


Figure S9.4 NLR of Hematological diseases subgroup analysis


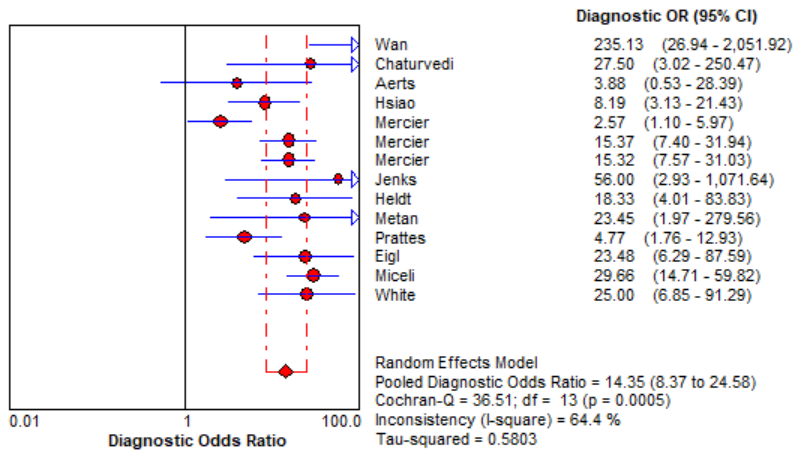


Figure S9.5 DOR of Hematological diseases subgroup analysis


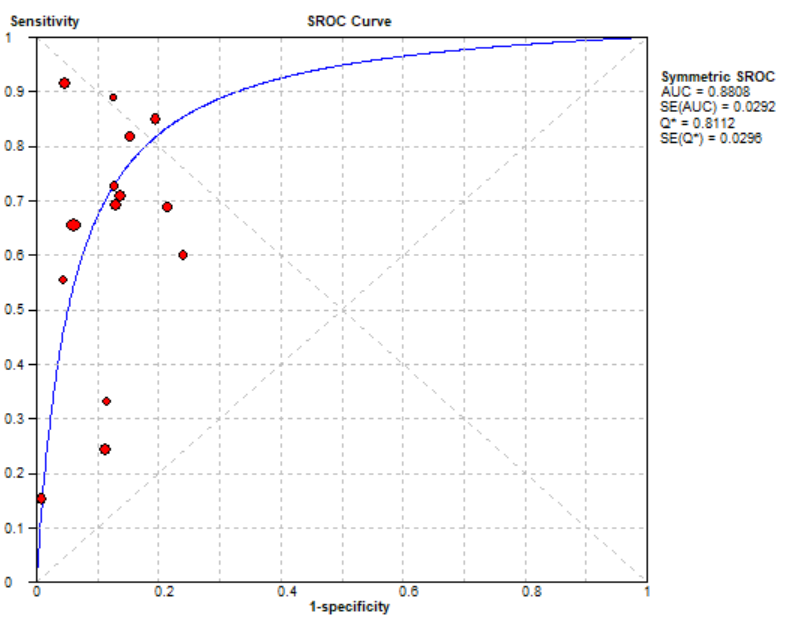


Figure S9.6 AUROC of Hematological diseases subgroup analysis

**Figure S10.1-10.6 Results of** **Respiratory diseases subgroup analysis**


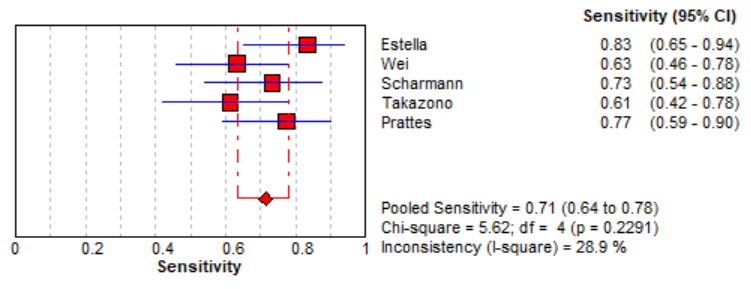


Figure S10.1 Sensitivity of Respiratory diseases subgroup analysis


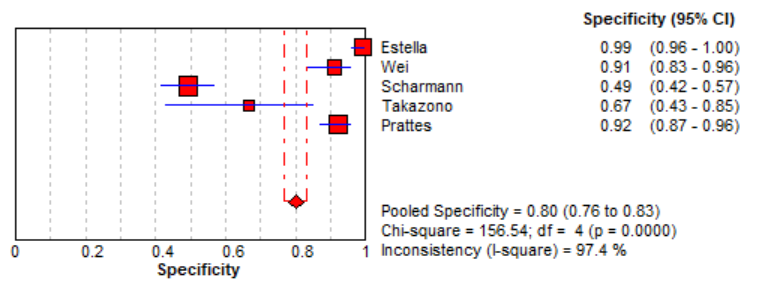


Figure S10.2 Specificity of Respiratory diseases subgroup analysis


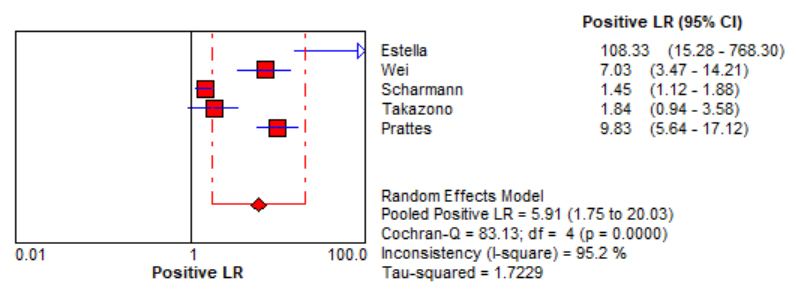


Figure S10.3 PLR of Respiratory diseases subgroup analysis


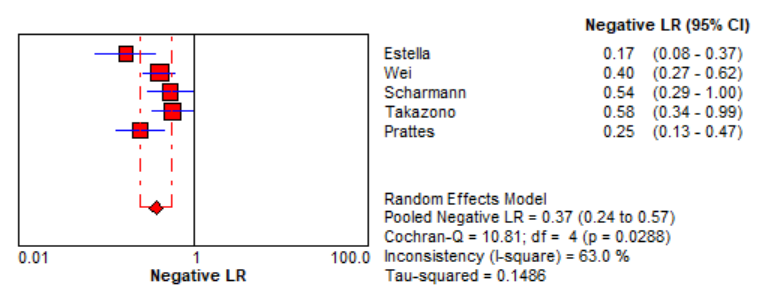


Figure S10.4 NLR of Respiratory diseases subgroup analysis


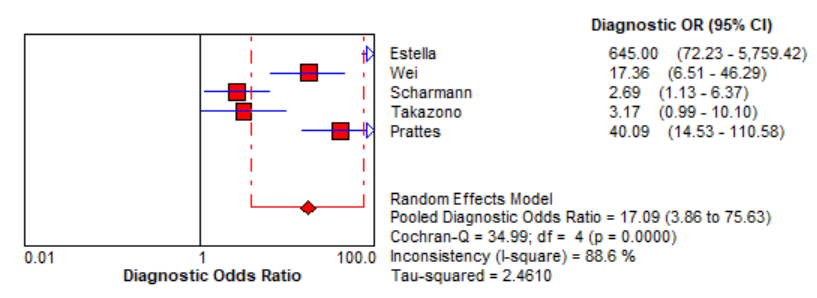


Figure S10.5 DOR of Respiratory diseases subgroup analysis


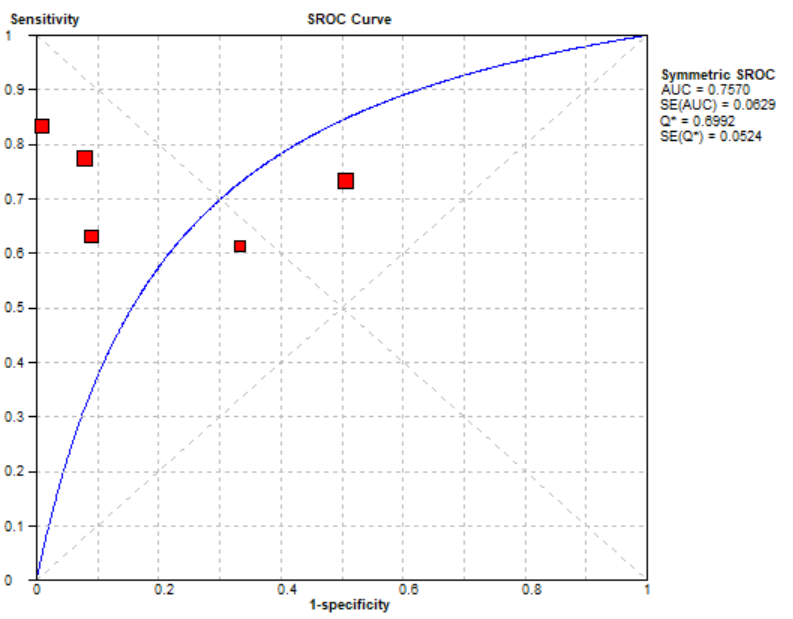


Figure S10.6 AUROC of Respiratory diseases subgroup analysis

**Figure S11.1-11.6 Results of** **Transplant patients subgroup analysis**


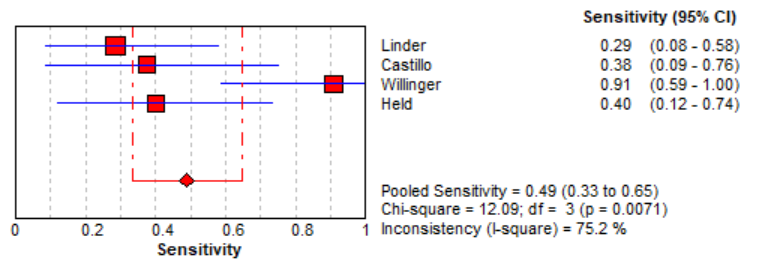


Figure S11.1 Sensitivity of Transplant patients subgroup analysis


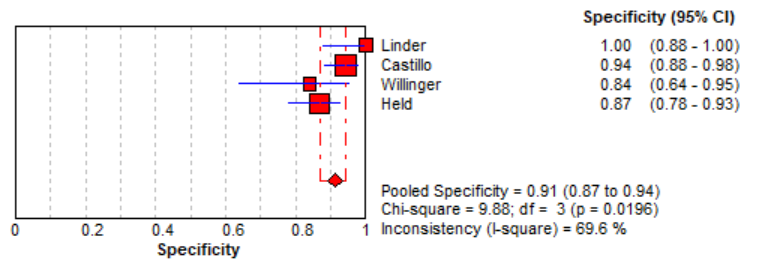


Figure S11.2 Specificity of Transplant patients subgroup analysis


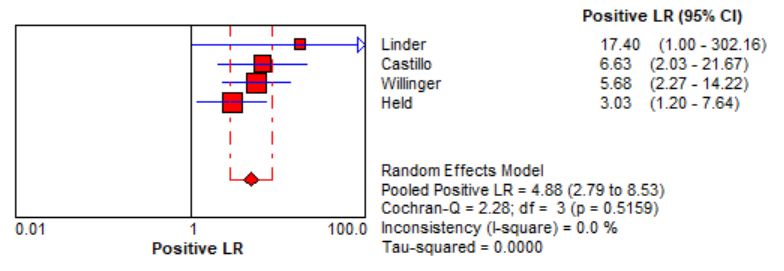


Figure S11.3 PLR of Transplant patients subgroup analysis


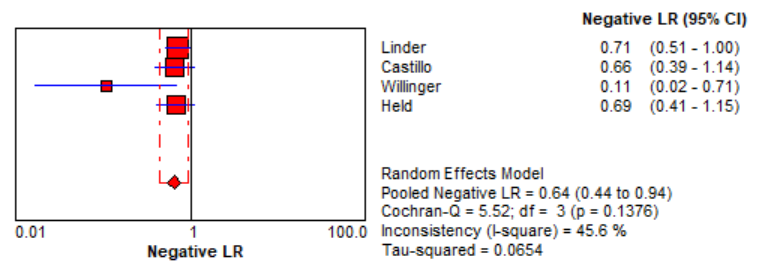


Figure S11.4 NLR of Transplant patients subgroup analysis


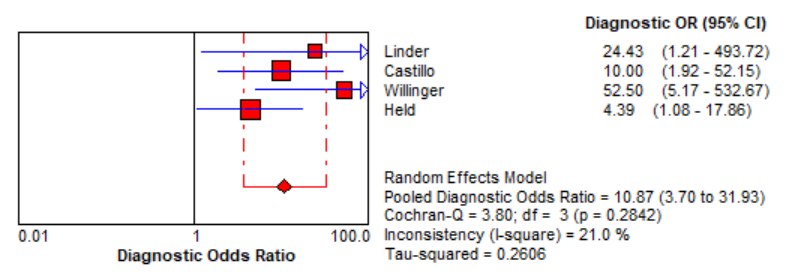


Figure S11.5 DOR of Transplant patients subgroup analysis


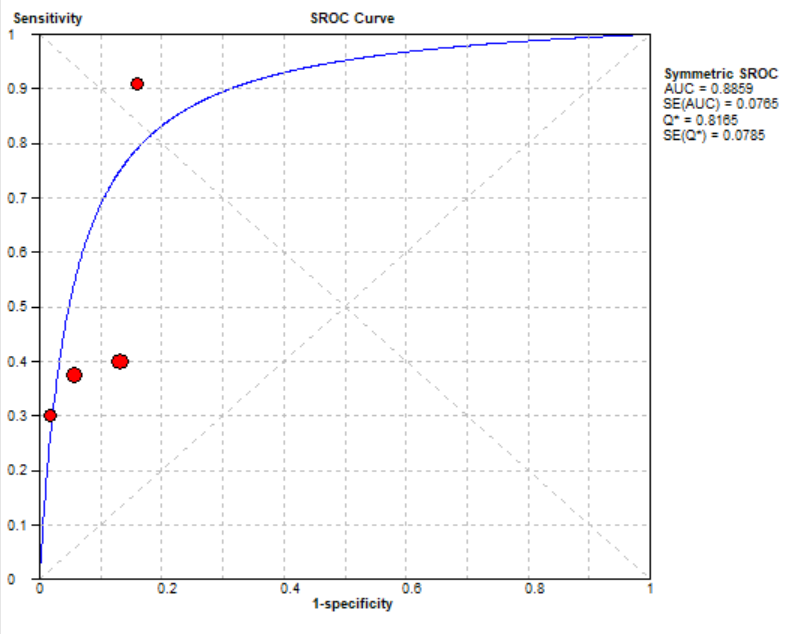


Figure S11.6 AUROC of Transplant patients subgroup analysis

**Figure S12.1-12.5 Results of** **Immunocompromised patients subgroup analysis**


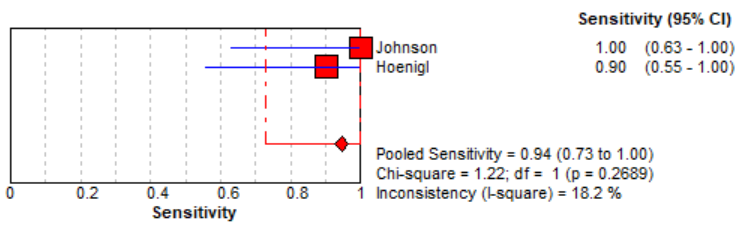


Figure S11.1 Sensitivity of Immunocompromised patients subgroup analysis


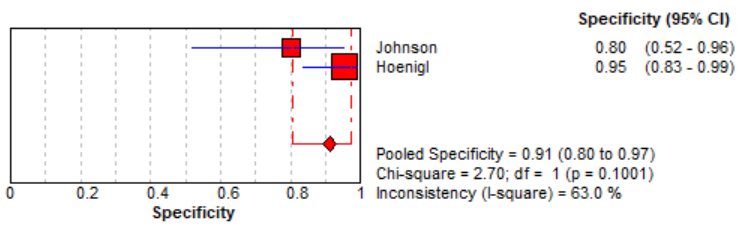


Figure S12.2 Specificity of Immunocompromised patients subgroup analysis


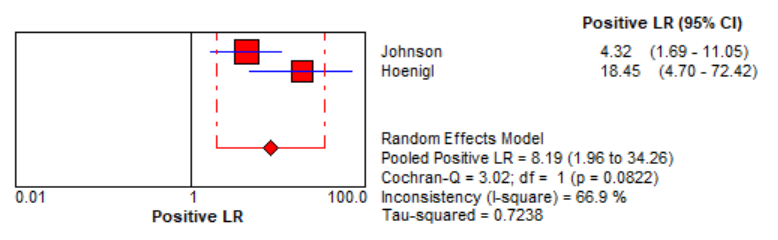


Figure S12.3 PLR of Immunocompromised patients subgroup analysis


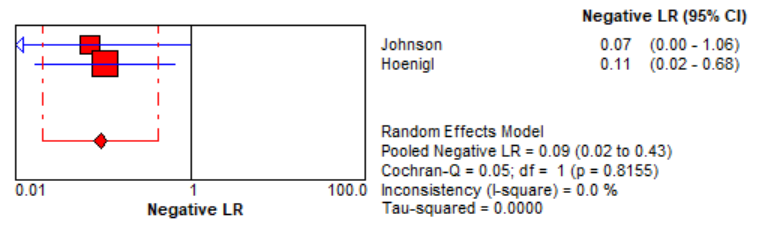


Figure S12.4 NLR of Immunocompromised patients subgroup analysis


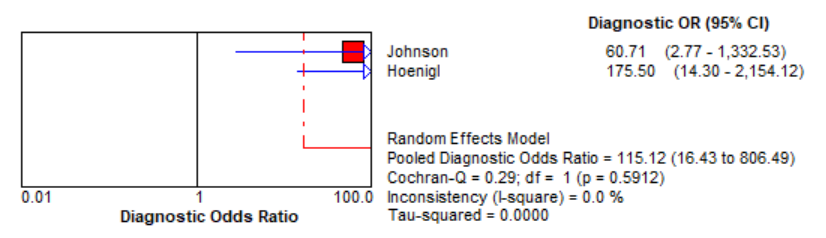


Figure S12.5 DOR of Immunocompromised patients subgroup analysis

**Note: AUROC of Immunocompromised patients subgroup analysis was not applicable (only 2 studies)**

**Publication bias test**

Figure S13. The results of Publication bias test
